# Synthesis of Dipyridylaminoperylenediimide–Metal Complexes and Their Cytotoxicity Studies

**DOI:** 10.3390/pharmaceutics14122616

**Published:** 2022-11-27

**Authors:** José Garcés-Garcés, Marta Redrado, Ángela Sastre-Santos, María Concepción Gimeno, Fernando Fernández-Lázaro

**Affiliations:** 1 Área de Química Orgánica, Instituto de Bioingeniería, Universidad Miguel Hernández de Elche, Avda. de la Universidad s/n, 03202 Elche (Alicante), Spain; 2 Departamento de Química Inorgánica, Instituto de Síntesis Química y Catálisis Homogénea (ISQCH), CSIC-Universidad de Zaragoza, C/ Pedro Cerbuna 12, 50009 Zaragoza, Spain

**Keywords:** perylenediimide, biological properties, cancer, cytotoxicity, metal complexes, silver, copper

## Abstract

A new family of perylenediimide (PDI) silver and copper complexes has been successfully synthesized by reacting *ortho*- and *bay*-substituted (dipyrid-2′,2″-ylamino)perylenediimide ligands with metal phosphine fragments. The coordination of the metal center did not reveal a significant effect on the photophysical properties, which are mainly due to the PDI ligands, and in some cases quenching of the luminescence was observed. The antiproliferative effect of the free perylenediimide ligands and the metalloPDI complexes against the cervix cancer cell line HeLa was determined by MTT assay. The free perylenediimide ligands exhibited a moderate cytotoxic activity, but the coordination of silver or copper to the dypyridylamino fragment greatly enhanced the activity, suggesting a synergistic effect between the two fragments. In attempts to elucidate the cellular biodistribution of the PDIs and the complexes, a colocalization experiment using specific dyes for the lysosomes or mitochondria as internal standards revealed a major internalization inside the cell for the metal complexes, as well as a partial mitochondrial localization.

## 1. Introduction

Perylenediimides (PDIs) are one of the most important dyes known today due to their outstanding electrooptical properties, high fluorescence quantum yields, strong absorption of visible light and huge versatility in their chemistry [1]. The properties of PDIs can be modified by the functionalization of the aromatic core in their different positions, namely *bay* positions (1, 6, 7 and 12) and *ortho* positions (2, 5, 8, and 11). Depending on the nature of the substituents on the aromatic core, their absorption profile, as well as the electron accepting/donating character of PDIs, can be drastically modified [2]. For these reasons, the appropriate design of the PDIs plays a key role in modulating their properties and molecular organization, which will lead to the optimal performance of the material.

Although PDIs are mainly used in optoelectronic applications [3] and material science [4], the number of biological studies using PDIs has been growing in the last few years [5,6,7], becoming promising molecules for biological investigation. Water-soluble PDIs have been used in biological applications due to their biocompatibility, photostability and optical absorption and emission properties. Synthetic strategies to gain water solubility consist either of the functionalization of PDIs with carbohydrate or PEG moieties, or the formation of charged PDI salts. The latter allows the interaction with other charged molecules present in the organism, such as DNA or proteins contained in the cell membrane [6]. On the other hand, PDI–carbohydrate conjugates are excellent candidates to label protein–carbohydrate interactions as glycodendrimers, presenting the potential to recognize carbohydrate–protein (lectin) interactions involved in key biological processes [8]. There are some examples on the use of PDIs as biological labels, such as the PDI–estradiol conjugate used to monitor the estrogen receptor by confocal microscopy [9]. PDIs bearing biotin and maleimide moieties have been used to target specific receptors, such as maltoporin [10]. PDIs substituted with galactose, mannose and fucose [11] have been used as chemosensors. PDIs are also involved in nanomedicine [12]; an example of this is a glycosacharide–PDI attached to maghemite nanoparticles tested as a dual imaging agent in magnetic resonance imaging. PDIs are also used in photodynamic therapy [13].

A simple strategy to modulate the properties of PDI systems using metallic fragments is the functionalization of the perylene core with coordinating donor groups that can act as ligands for metal complex formation. Thus, PDI derivatives containing palladium [14] and gold [15] complexes have been synthesized for electrooptical applications and to prepare Langmuir films, respectively. A PDI self-assembly to construct silver nanohybrids with enhanced visible-light photocatalytic antibacterial effects has also been described [16]. Additionally, iridium-containing PDI complexes [17] have been used as organic light-emitting devices (OLEDs). Moreover, functionalization of PDIs with electron-donating groups to coordinate Cu^+^, Cu^2+^ and Fe^3+^ allowed the development of photo-induced electron transfer systems with the aim to emulate the photosynthetic process [18]. On the other hand, the supramolecular chemistry of metallo-PDIs is an emerging research area; thus, metallo-cages based on PDIs [19] show high fluorescence quantum yields and the ability to host polycyclic aromatic hydrocarbon, such as pyrene or triphenylene. Biological properties of metal complex derivates of PDIs have been investigated too, as in the case of a PDI functionalized with phenanthroline moieties to coordinate ruthenium (II) [20], which was studied for photodynamic therapy. However, almost no studies have been published on PDI metallocomplexes as anticancer or theranostic agents.

One of the most promising metals that presents several biological properties is silver. For many years, silver complexes have been used for antiseptic, antibacterial or anti-inflammatory applications [21], taking advantage of their low cytotoxicity [22]. Silver organometallic complexes have also been studied as anticancer agents, as many silver (I) complexes have been found to exhibit a greater cytotoxic activity than cisplatin, with relatively low toxicity and greater selectivity toward cancer cells [23,24]. Cell death via apoptosis and depolarization of the mitochondrial membrane potential are the most accepted mechanisms of this anticancer activity [24,25,26,27,28]. On the other hand, copper salts have been less studied as drugs. Copper is an essential metal for organisms, playing a key role in numerous cellular processes. In particular, copper is the cofactor in important metalloenzymes for the mitochondrial metabolism and in detoxification of radical oxygen species (ROS). Copper complexes are potent topoisomerase inhibitors, the redox activity of [Cu(I)/Cu(II)] being one of the principal causes of cytotoxicity [29].

In this context, in order to study the synergistic influence of PDI ligands and their metal complexes as anticancer agents, we present here the synthesis and in vitro studies of a new family of silver and copper complexes derived from *ortho*- and *bay*-substituted (dipyrid-2′,2″-ylamino)perylenediimides, testing the free perylenediimide ligand and the metalloPDI complexes against HeLa cell line.

## 2. Materials and Methods

### 2.1. Synthetic Procedure

All chemicals were reagent grade, purchased from commercial sources, and were used as received unless otherwise specified. Column chromatography was performed on SiO_2_ (40–63 lm) (Carlo Erba, Barcelona, Spain). TLC plates coated with SiO_2_ 60F254 were visualized under UV light (Macherey-Nagel, Düren, Germany).

^1^H NMR and ^31^P{^1^H} NMR spectra were recorded at room temperature on a BRUKER AVANCE 400 spectrometer (Bruker, Billerica, MA, USA) (^1^H, 400 MHz) or on a BRUKER AVANCE II 300 spectrometer (^1^H, 300 MHz), with chemical shifts (ppm) reported relative to the solvent peaks in the ^1^H spectra or external 85% H_3_PO_4_ in ^31^P{^1^H} of the deuterated solvent. CD_2_Cl_2_ and CDCl_3_ were used as the deuterated solvents (Euroisotop, Saint-Aubin, France). Chemical shifts were reported in the *δ* scale relative to residual CH_2_Cl_2_ (5.32 ppm) and TMS (0 ppms). NMR were recorded at ambient probe temperature. UV-vis spectra were recorded with a Perkin Elmer Lambda 365 spectrophotometer (Tres Cantos, Madrid, Spain) in CHCl**_3_** solutions in the range 250 to 800 nm. Fluorescence spectra were recorded with a HORIBA scientific SAS spectrophotometer (Palaiseau, France) in CHCl_3_ solutions in the range 250 to 800 nm. High-resolution mass spectra were obtained from a Bruker Microflex LRF20 (Bruker, Boston, MA, USA) matrix-assisted laser desorption/ionization time of flight (MALDI-TOF), using dithranol as a matrix. IR spectra were recorded with a Nicolet Impact 400D spectrophotometer (ThermoFisher, Scienfic) in KBr in the range 4000–400 cm**^−1^**.

The starting material [Ag(OTf)(PPh_3_)] [30] was prepared according to published procedures. All other reagents and solvents were commercially available (Merk Life Science, Madrid, Spain).

#### 2.1.1. Synthesis of *N*,*N′*-Diethylpropyl-1-(dipyrid-2′,2″-ylamine)perylene-3,4:9,10-tetracarboxydiimide (**PDI-2**)

*N*,*N*′-Diethylpropyl-1-bromoperylene-3,4:9,10-tetracarboxydiimide **PDI-1** (50 mg, 0.06 mmol), 2,2′-dipyridylamine (31 mg, 0.181 mmol), cesium carbonate (70 mg, 0.21 mmol) and 1,1′-bis[(diphenylphosphino)ferrocene]dichloropalladium (II) (4.4 mg, 0.0054 mmol) were added to a two-neck round-bottom flask and flushed with nitrogen for 30 min. Then, dry toluene (8 mL) was injected and stirred at 80 °C for 24 h under nitrogen atmosphere. The cooled mixture was extracted with dichloromethane and washed with water. The organic phase was dried over anhydrous sodium sulfate, filtered, and evaporated. Purification was carried out by silica gel column chromatography (toluene:acetone, 20:1), yielding 30 mg (70%) of **PD-2** as a purple solid. ^1^H NMR (300 MHz, CD_2_Cl_2_) δ 9.24 (d, J = 8.3 Hz, 1H, H_g_), 8.67-8.62 (m, 4H, H_b–e_), 8.38 (bs, 1H, H_a,_), 8.34 (d, J = 8.3 Hz, 1H, H_f_) 8.22 (ddd, J = 4,93, 1.08, 0.89 Hz, 2H, H_k_), 7.58 (dddd, J = 6.44, 5.32, 1.98, 1–95 Hz, 2H, H_m_), 7.20 (d, J =7.36, 2H, H_n_), 6.97 (ddd, J = 7.26, 4.91, 0.95 Hz, 2H, H_l_), 5.04–4.92 (m, 2H, H_h_), 2.28–2.10 (m, 4H, H_h_), 1.96–1.80 (m, 4H, H_i_), and 0.87 ppm (dt, J = 13.8, 7.5 Hz, 12 H, H_j_). ^13^C NMR (75 MHz, CDCl_3_) δ 155.9, 149.1, 143.1, 138.1, 134.8, 134.4, 132.9, 129.3, 129.1, 129.0, 128.3, 128.2, 126.7, 126.5, 125.4, 123.6, 122.7, 119.5, 115.8, 57.8, 57.6, 25.1, 25.1, 11.4 and 11.41 ppm. FT-IR (KBr): 3457, 2962, 2872, 1704, 1650, 1593, 1458, 1426, 1401, 1332, 1242, 1193, 1144, 1086, 854, 813, 776, 743, and 694 cm*^−^*^1^. UV-Vis (CHCl_3_) λ _max_/nm (log ε): 561 (4.48), 480 (4.55). HR-MALDI-TOF *m*/*z* [M^+^] calc. for C_44_H_37_N_5_O_4_: 699.285, found: 699.280.

#### 2.1.2. Synthesis of *N*,*N*′-Diethylpropyl-2,5,8,11-tetra(dipyrid-2′,2″-ylamine)perylene-3,4:9,10 tetracarboxydiimide (**PDI-6**)

*N*,*N´*-Diethylpropyl-2,5,8,11-tetrabromoperylene-3,4:9,10-tetracarboxydiimide **PDI-5** (50 mg, 0.06 mmol), 2,2′-dipyridylamine (123 mg, 0.72 mmol), cesium carbonate (273 mg, 0.84 mmol) and 1,1′-bis[(diphenylphosphino)ferrocene]dichloropalladium (II) (21 mg, 0.021 mmol) were added to a two-neck round-bottom flask and flushed with nitrogen for 30 min. Then, dry toluene (30 mL) was injected and stirred at 80 °C for 24 h under nitrogen atmosphere. The cooled mixture was extracted with dichloromethane and washed with water. The organic phase was dried over anhydrous sodium sulfate, filtered and evaporated. Purification was carried out by silica gel column chromatography (toluene:acetone, 20:1) yielding 30 mg (69%) of **PDI-6** as a purple solid. ^1^H NMR (300 MHz, CD_2_Cl_2_) δ 8.15 (d, J = 3.7 Hz, 8H, H_e_), 7.98 (s, 4H, H_a_), 7.61–7.55 (m, 8H, H_g_), 7.16 (d, J = 8.3 Hz, 8H, H_h_), 6.93 (dd, J = 6.9, 5.2 Hz, 8H, H_f_), 4.41–4.31 (m, 2H, H_b_), 1.58–1.43 (m, 4 H, H_c_), 1.36–1.22 (m, 4H, H_c_) and 0.33 ppm (t, J = 7.4 Hz, 12H, H_d_). ^13^C NMR (75 MHz, CDCl_3_) δ 162.3, 157.3, 148.5, 148.2, 138.0, 134.9, 133.3, 126.7, 123.6, 119.3, 119.1, 117.7, 57.7, 24.9 and 11.6 ppm. FT-IR (KBr): 3457, 2962, 2929, 2872, 1704, 1650, 1593, 1458, 1426, 1401, 1332, 1242, 1193, 1144, 1086, 854, 813, 776, 743, and 694 cm*^−^*^1^.UV-Vis (CHCl_3_) λ _max_/nm (log ε): 536 (4.81), 496 (4.78). HR-MALDI-TOF *m*/*z* [M^+^] calc. for C_74_H_58_N_14_O_4_: 1206.477, found: 1206.475.

#### 2.1.3. Synthesis of Complex **PDI-3**

**PDI-2** (10 mg, 0.014 mmol) was dissolved in DCM (2.5 mL) and then [Ag(OTf)PPh_3_] (7.41 mg, 0.014 mmol) was added. The mixture was stirred at room temperature for 1 h and then the solvent was evaporated until dryness, yielding 14.97 mg (100%) of **PDI-3** as a purple solid. No purification step was needed. ^1^H NMR (300 MHz, CD_2_Cl_2_) δ 9.20 (d, J = 8.3 Hz, 1 H, H_g_), 8.74–8.61 (m, 4H_b–e_),8.39 (s, 1H, H_a_), 8.29 (d, J = 4.07 Hz, 2H, H_k_), 8.24 (d, J =8.3 Hz, 2H, H_f_), 7.67 (dddd, J = 8.38, 7.33, 1.89, 1.89 Hz, 2 H, H_m_), 7.46–7.29 (m, 15 H, Ar-Ph), 7.24 (d, J = 8.36 Hz, 2H, H_n_), 6.97 (dd, J = 6.69, 5.37 Hz, 2H, H_l_), 5.07–4.91 (m, 2H, H_h_), 2.26–2.10 (m, 4 H, H_i_), 1.98–1.81 (m, 4 H, H_i_), and 0.88 ppm (dt, J = 14.9, 7.5 Hz, 12 H, H_j_). ^31^P NMR (121 MHz, CD_2_Cl_2_) δ 16.7 and 12.2 ppm. FT-IR (KBr): 3473, 2962, 2365, 1699, 1654, 1593, 1434, 1328, 1242, 1021, 821, 756, 702, and 629 cm*^−^*^1^ UV-Vis (CHCl_3_) λ _max_/nm (log ε): 561 (4.91), 481 (4.75) and 274 (4.29). HR-MALDI-TOF *m*/*z* [M^+^] calc. for C_62_H_52_AgN_5_O_4_P: 1069.288, found: 1069.282.

#### 2.1.4. Synthesis of Complex **PDI-4**

**PDI-2** (20 mg, 0.029 mmol) was dissolved in DCM (2.5 mL) and then [Cu(NO_3_)(PPh_3_)_2_] (18.85 mg, 0.029 mmol) was added. The mixture was stirred at room temperature for 1 h and then the solvent was evaporated until dryness, yielding 37 mg (100%) of **PDI-4** as a purple solid. No purification step was needed. ^1^H NMR (300 MHz, CD_2_Cl_2_) δ 9.28 (d, J = 8.3 Hz, 1H, H_g_), 8.70–8.64 (m, 4H, H_b–e_), 8.48 (s, 1H, H_a_), 8.42 (d, J = 8.30 Hz, 1H, H_f_) 8.31–(dd, J = 4.66, 1.1 Hz, 2 HH_k_), 7.62–7.57 (m, 2H, H_m_), 7.44–7.2 (m, 30 H, Ar-Ph), 7.19 (d, J = 8.3 Hz, 2H, H_n_), 6.99 (dd, J = 6.8, 5.1 Hz, 2H, H_l_), 5.12–5.0 (m, 2H, H_h_), 2.38–2.16 (m, 4H, H_i_), 2.03–1.86 (m, 4H, H_i_), and 0.93 ppm (dt, J = 10.1, 7.5 Hz, 12 H, H_j_). ^31^P NMR (121 MHz, CD_2_Cl_2_) δ −0.47 ppm. FT-IR (KBr): 3052, 2958, 2925, 2872, 1691, 1654, 1597, 1458, 1433, 1405, 0380, 1331, 1249, 1192, 1090, 812, 738, 698, and 500 cm*^−^*^1^. UV-Vis (CHCl_3_) λ _max_/nm (log ε). 561 (4.91), 481 (4.75) and 274 (4.29). MALDI-TOF *m*/*z* [M-PPh*_3_*]*^+^* calc. for C_80_H_67_CuN_5_O_4_P_2*–*_PPh_3_: 1024.305, found for C_80_H_67_CuN_5_O_4_P_2_*–*PPh_3_: 1024.340.

#### 2.1.5. Synthesis of Complex **PDI-7**

**PDI-6** (10 mg, 8.28·10*^−^*^3^ mmol) was dissolved in DCM (2.5 mL) and then [Ag(OTf)PPh_3_] (17.20 mg, 0.033 mmol) was added. The mixture was stirred at room temperature for 1 h and then the solvent was evaporated until dryness, yielding 22 mg (99%) of **PDI-7** as a purple solid. No purification step was needed. ^1^H NMR (300 MHz, CD_2_Cl_2_) δ 8.16 (bs, 12H, H_a_, H_e_), 7.58 (t, J = 7.1 Hz, 8H, H_g_), 7.49–7.34 (m, 60H, Ar-Ph) 7.10 (d, J = 8.2 Hz, 8H, H_h_), 6.93–6.89 (m, 8 H, H_f_), 4.25–4.20 (m, 2H, H_b_), 1.40–1.27 (m, 4H, H_c_), 1.15–1.06 (m, 4H, H_c_), and 0.16–0.09 ppm (m, 12H, H_d_). ^31^P NMR (121 MHz, CD_2_Cl_2_) δ 18.38 and 12.29 ppm. FT-IR (KBr): 3052, 2966, 2921, 2860, 1699, 1663, 1589, 1462, 1430, 1377, 1332, 1274, 1254, 1180, 1091, 1050, 776, 474, 694, 657, and 523 cm*^−^*^1^. λ _max_/nm (log ε): 536 (4.62), 271 (4.70).

#### 2.1.6. Synthesis of Complex **PDI-8**

**PDI-6** (10 mg, 8.28·10**^−^**^3^ mmol) was dissolved in DCM (2.5 mL) and then [Cu(NO_3_)(PPh_3_)_2_] (21.54 mg, 0.033 mmol) was added. The mixture was stirred at room temperature for 1 h and then the solvent was evaporated until dryness, yielding 29 mg (99%) of **PDI-8** as a purple solid. No purification step was needed. ^1^H NMR (300 MHz, CD_2_Cl_2_) δ 8.13 (bs, 8H, H_e_), 7.99 (bs, 4H, H_a_), 7.60–7.55 (m, 8 H. H_g_), 7.42–7.28 (m, 120H, Ar-Ph), 7.56 (bs, 8H, H_h_), 6.91 (bs, 8H, H_f_), 4.42–4.37 (m, 2H, H_b_), 1.61–1.46 (m, 4H. H_c_), 1.36–1.29 (m, 4H, H_c_), and 0.35 ppm (t, J = 7.3 Hz, 12 H, H_d_). ^31^P NMR (121 MHz, CD_2_Cl_2_) δ −0.48 ppm. FT-IR (KBr): 3436, 3052, 3003, 2958, 2921, 2361, 1691, 1654, 1585, 1467, 1434, 1385, 1344, 1274, 1209, 1091, 1025, 988, 735, 694, 523, and 506 cm**^−^**^1^. UV-Vis (CHCl_3_) λ_max_/nm (log ε): 536 (4.66), 274 (4.66).

### 2.2. Cell Culture

HeLa (cervical cancer) cell line (from ATCC, USA) was routinely cultured in high-glucose DMEM medium supplemented with 5% fetal bovine serum (FBS), L-glutamine and penicillin/streptomycin (hereafter, complete medium) at 37 °C in a humidified atmosphere of 95% air/5% CO_2_.

### 2.3. Cell Viability Assays

The MTT-reduction assay was used to analyse cell metabolic activity as an indicator of cell sensitivity to compounds **PDI-2** to **PDI-4**, and **PDI-6** to **PDI-8** in the HeLa cell line. A total of 6000 cells/well were seeded in 96-well plates (100 µL/well) and allowed to attach for 24 h prior to addition of compounds. The complexes were dissolved in DMSO and added to cells in concentrations ranging from 0.2 to 50 µM in quadruplicate. Cells were incubated with our compounds for 24 h, then 10 µL of MTT (5 mg/mL in PBS) were added to each well and plates were incubated for 2 h at 37 °C. Finally, the culture medium was removed and DMSO (100 µL/well) was added to dissolve the formazan crystals. The optical density was measured at 550 nm using a 96-well multiscanner autoreader (ELISA) and IC_50_ was calculated. Each compound was analyzed at least in three independent experiments.

### 2.4. Cytotoxicity Assays

Apoptotic cell death was determined by measuring phosphatidyl-serine exposure on cell surface in HeLa cells. A total of 60,000 cells/well were seeded in 12-well plates (1 mL/well) and left overnight to be attached to the bottom. Cells were treated for 24 h with complexes **PDI-3** and **PDI-4** at IC_50_ and 2·IC_50_ concentrations, respectively, in duplicate. After treatment, cells were trypsinised and resuspended in 50 µL of a mixture of Anexin-binding buffer (ABB; 140 mM NaCl, 2.5 mM CaCl_2_, 10 mM HEPES/NaOH pH 7.4), FITC-conjugated Annexin V and incubated at room temperature in the dark for 15 min. Finally, cells were diluted to 250 μL with ABB and a total of 10,000 cells were acquired on a FACSCalibur flow cytometer (BD Biosciences, Franklin Lakes, NJ, USA). Cell death was analyzed using CellQuest Pro (BD Biosciences, Franklin Lakes, NJ, USA), FlowJo 7.6.1 (Becton Dickinson (BD), Franklin Lakes, NJ, USA) and GraphPad Prism 5 (GraphPad Software, San Diego, CA, USA) software.

### 2.5. Fluorescence Confocal Microscopy

A total of 10^4^ HeLa cells/well were seeded in complete medium in µ-slide 8 well (ibiTreat) (300 µL/well) and left 24 h to be attached to the bottom. Then, 200 µL of culture medium were removed and 100 µL of a solution of species **PDI-2**, **-3**, **-4** and **-8** were added to a final concentration of 2 µM. The compounds were incubated with the cells for 2 h. Thereafter, MitoTracker Green (MTG) or LysoTracker Green (LTG) was added to a final concentration of 75 nM and 500 nM, respectively, and it was incubated with the cells for 30–45 min at room temperature. Eventually the medium was replaced with fresh medium without phenol red. Images were collected in a sequential mode in a FluoView FV10i (Olympus, Shinjuku, Japan) confocal microscope with a 40 oil immersion lens, a line average of 4, and a format of 1024 × 1024 pixels using excitation wavelength of either 488 or 561 nm. The confocal pinhole was 1 Airy unit. Images were analysed with FV10-ASW 3.1. Viewer software.

### 2.6. Cell Morphology Analysis

Alterations in cell morphology and behavior as a consequence of the exposure to complexes **PDI-3** and **PDI-4** were analyzed using an inverted microscope Olympus IX71 Inverted. A total of 10,000 cells/well were seeded in 12-well plates (1 mL/well) and left overnight to be attached to the bottom. Thereafter, cells were treated for 24 h with complexes at IC_50_ and 2·IC_50_ concentrations, respectively, in duplicate.

## 3. Results and Discussion

### 3.1. Synthesis of PDIs and Metal Complexes

**Scheme 1:**
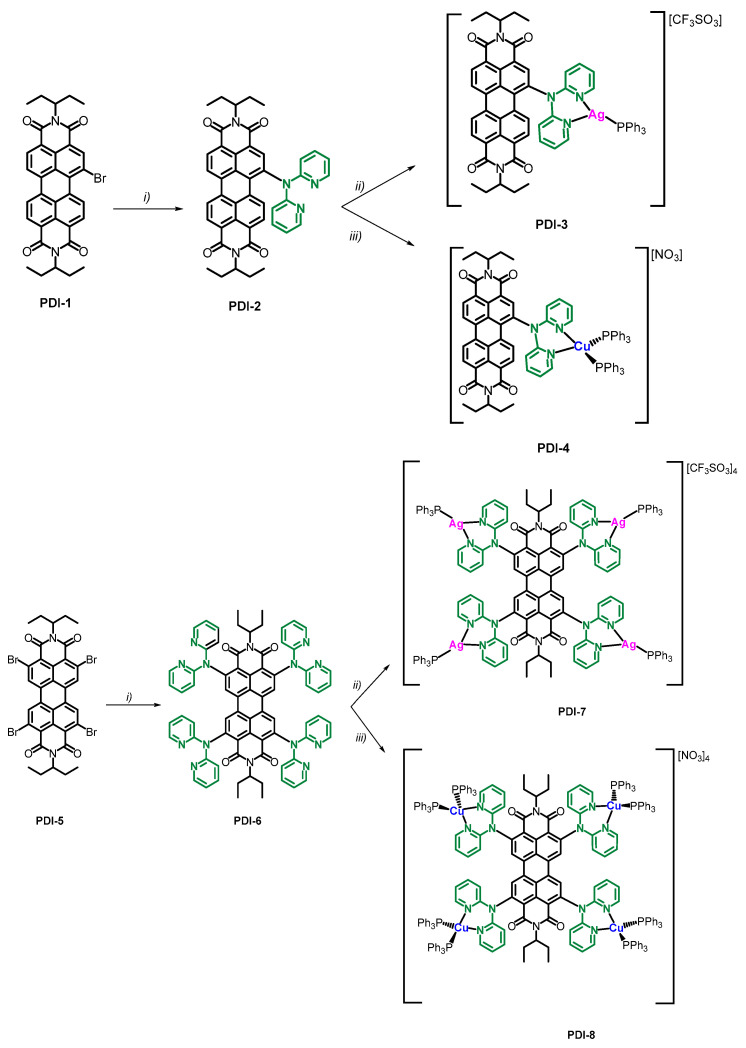
Reagents and conditions: (**i**) 2,2′-dipyridylamine, [Pd(dppf)_2_Cl_2_]_,_ DCM, Cs_2_CO_3_, toluene, 24 h, 80 °C, nitrogen; (**ii**) [Ag(OTf)(PPh_3_], DCM, 1 h, rt; (**iii**) [CuNO_3_(PPh_3_)_2_], DCM, 1 h, rt.

**PDI-1** and **PDI-5** were synthesized as described in the literature [31,32,33]. **PDI-2** and **PDI-6** were synthesized for the first time using a Buchwald–Hartwig cross-coupling reaction [34,35]. Thus, bromo **PDI-1** and tetrabromo **PDI-5** were reacted with 2,2′-dipyridylamine in the presence of bis[(diphenylphosphino)ferrocene]dichloropalladium(II) in a basic media to obtain **PDI-2** and **PDI-6** with 70% and 69% yield, respectively (Scheme 1). Both PDIs were fully characterized by spectroscopic and spectrometric methods (see the Supporting Information).

The addition of either [Ag(OTf)(PPh_3_)] or [CuNO_3_(PPh_3_)_2_] to a solution of **PDI-2** or **PDI-6** in a 1:1 or 4:1 molar ratio, respectively, led to the formation of the phosphine silver (I) complexes **PDI-3** and **PDI-7**, and the phosphine copper(I) complexes **PDI-4** and **PDI-8** (Scheme 1). In all cases, complexation reactions were quantitative, and no purification steps were required.

**Figure 1:**
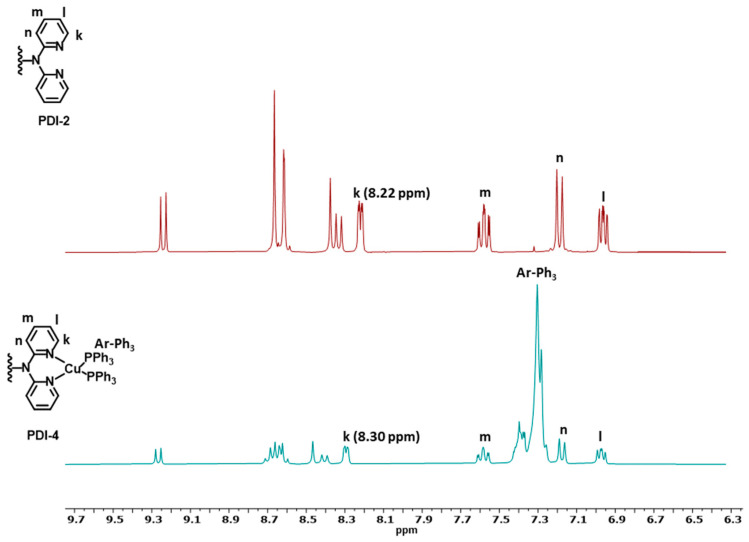
Part of ^1^H NMR of **PDI-2** (red) and **PDI-4** (blue) in CD_2_Cl_2_ at 25 °C. Letters over the NMR signals refer to the different hydrogen atoms of the ligands (see figures in the inset).

Figure 1 shows the comparison between **PDI-2** and the mono-copper complex **PDI-4**. We can observe that all aromatic protons of the pyridyl group were weakly deshielded in **PDI-4** in comparison with **PDI-2** due to copper complexation, Hk being the most affected. At 7.44–7.26 ppm we found the signal attributed to the phosphine groups integrating for 30 H corresponding to two phosphine groups. The mono-silver complex **PDI-3** followed the same pattern (see Supporting Information Figure S11).

**Figure 2:**
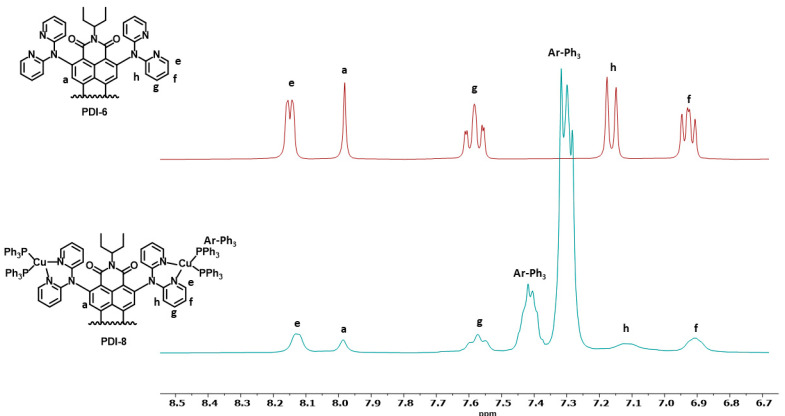
Part of ^1^H NMR of **PDI-6** (red) and **PDI-8** (blue) in CD_2_Cl_2_ at 25 °C.

Figure 2 shows the comparison between **PDI-6** and the tetra-copper complex **PDI-8**. In this case, all signals were broadened, possibly because of the rotation of the four substituted dipyridylamino metallo units in **PDI-6–8.** Additionally, we can observe the resonance corresponding to the aromatic protons of the phosphine groups between 7.28 and 7.45 ppm integrating for 120 H, indicating the presence of two phosphine groups for each copper unit. The same broadening effect was observed in the tetra-silver complex **PDI-7**. In this case, an integration of 60 H of the phosphine signals agrees with the existence of four triphenylphosphine units (see Supporting Information Figure S17).

**Figure 3:**
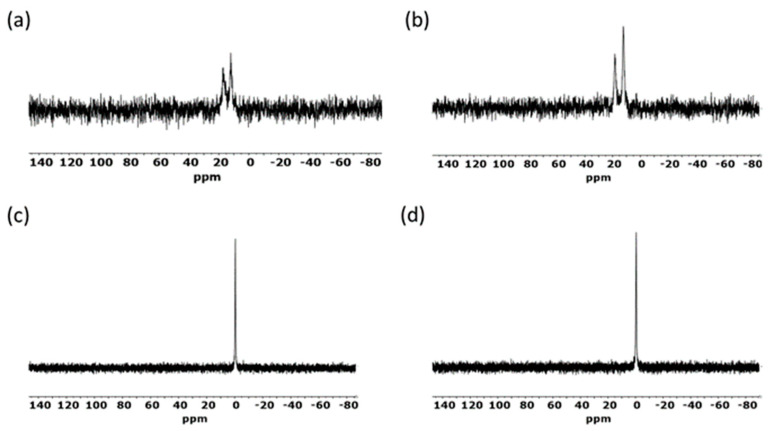
^31^P{^1^H} NMR spectra of PDI metal complexes (**a**) **PDI-3**, (**b**) **PDI-7**, (**c**) **PDI-4**, (**d**) **PDI-8** in CD_2_Cl_2_ at 25 °C.

In the case of silver complexes, **PDI-3** and **PDI-7**, the ^31^P{^1^H} NMR spectrum showed a broad doublet at 12.02 and 17.12 ppm, respectively, due to the coupling of the phosphorus atom with the two silver isotopes ^109^Ag and ^107^Ag, corresponding to the average coupling. The copper complexes **PDI-4** and **PDI-8** showed a signal at −0.5 ppm in the ^31^P{^1^H} NMR spectrum in agreement with the presence of equivalent phosphorus atoms (Figure 3).

### 3.2. Absorption and Fluorescence Studies

The presence of N atoms in **PDI-2** and **PDI-6** quenches the fluorescence in these PDIs. Thus, while **PDI-1** has a fluorescence quantum yield of 91%, it drops to 35% in **PDI-2**. For **PDI-6,** fluorescence is completely quenched (see Supporting Information, Figure S9). In the case of the mono-substituted complexes **PDI-3** and **PDI-4**, the fluorescence quantum yields were 49 and 57%, respectively, being higher than in the precursor **PDI-2**.

**Figure 4:**
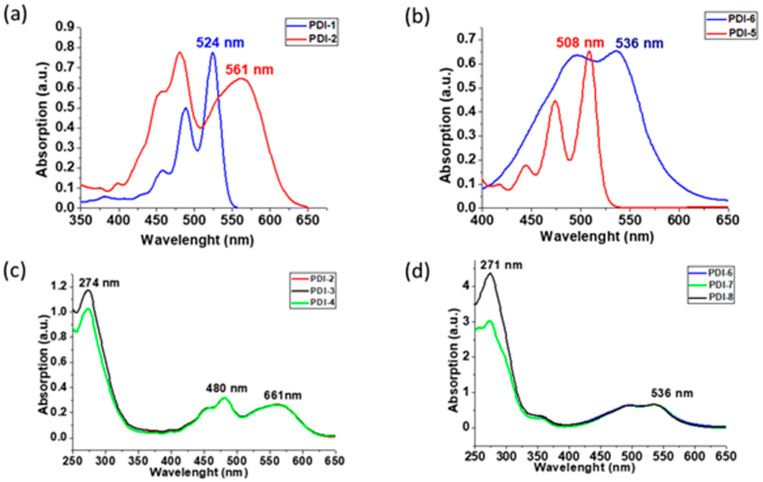
Normalized absorption in CHCl_3_ as solvent at 25 °C of (**a**) **PDI-1** and **PDI-2**, (**b**) **PDI-5** and **PDI-6**, (**c**) **PDI-2**, **PDI-3** and **PDI-4**, and (**d**) **PDI-6**, **PDI-7** and **PDI-8**.

The absorption spectrum of **PDI-2** in chloroform solution changed totally in respect to the precursor **PDI-1**, which located its maximum at 524 nm, typical for a *bay*-substituted PDI with electron withdrawing groups. **PDI-2** showed a broad band at 561 nm, which corresponds to the charge transfer from the dipyridylamine moieties to the perylene core (Figure 4a). On the other hand, functionalization of **PDI-5** in the *ortho* positions with dipyridylamine induced a dramatic bathochromic shift, changing the maximum of **PDI-5** located at 508 nm to 536 nm in **PDI-6** (Figure 4b).

The UV-vis spectra in chloroform of the four metal complexes, **PDIs-3–4** and **PDIs-7–8**, show new absorption bands at 274 and 271 nm, attributed to the phenyl groups of the phosphine moieties, while the absorption band attributed to the perylene core remains unaffected after complexation reaction (Figure 4c,d).

### 3.3. Antiproliferative Studies

Antiproliferative studies were carried out for all compounds against human cervical carcinoma (HeLa) cancer cell line using the MTT assay [36], and the results are shown in Table 1. We tested the stability of all compounds in DMSO, the medium used in the in vitro assays, ^1^H NMR, corroborating that they remained stable after a few days. In addition, the stability of the complexes in the biological media (Phosphate Buffer Solution PBS with 5% of DMSO) was measured using Uv-Vis spectra. The spectra measured at 0 and 24 h showed that the PDI and the corresponding silver complexes remained stable in solution (see Figure S23). However, some differences in the high energy absorptions appeared for the copper complex **PDI-8** and, consequently, we can not discard that this complex could dissociate some PPh**_3_** ligands in DMSO or biological solutions. The effect of this dissociation in the cytotoxic properties will be studied in due course.

The data show that the starting **PDI-2** and **PDI-6** ligands were moderately active, with half minimum inhibitory concentrations (IC_50_) of 11.51 ± 0.86 and 10.54 ± 0.82 µM, respectively. Coordination of the silver or copper fragments greatly enhanced the cytotoxic activity, and the final complexes exhibited IC_50_ values in the low micromolar range. Analyzing the results as a function of the metal, a clear tendency was not observed because for **PDI-3** and **PDI-4**, the silver complex presented a slightly higher activity than the copper one, but the opposite result was obtained for **PDI-7** and **PDI-8**. However, these differences may not be significant, as the complexes in general exhibited excellent activity.

### 3.4. Morphological Appearance and Cell Death Mechanism

**Figure 5:**
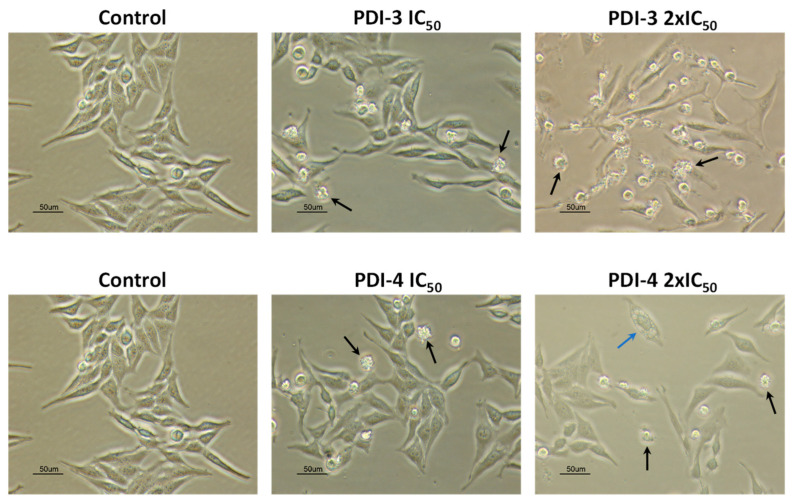
Phase contrast microscopy images of HeLa cells untreated (control) and treated with **PDI-3** and **PDI-4** at concentrations equal to the IC_50_ and 2 × IC_50_ µM for 24 h. Black arrows point to apoptotic cells and blue arrow to necrotic cells.

Cellular behavior and morphological alterations of HeLa cells after exposure to the complexes were analyzed under an inverted microscope. Untreated cells were healthy, grew exponentially and exhibited their characteristic morphology, whereas the cells treated with the silver and copper compounds at concentrations about and double the IC_50_ showed alterations in the morphology (Figure 5). It is noticeable that for copper compound **PDI-4**, the formation of apoptotic death cells was observed, whereas at higher concentration some of the cells were greatly disturbed and presented a necrotic morphology. For the silver compound **PDI-3**, an apoptotic cell death envisaged an even higher concentration.

**Figure 6:**
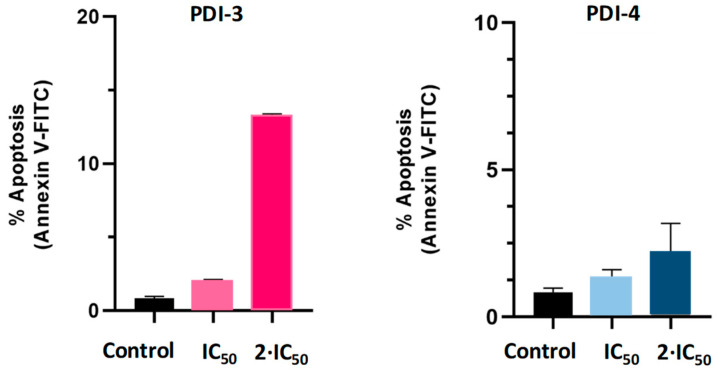
Cytotoxicity assays of compounds **PDI-3** and **PDI-4** incubated in HeLa cells for 24 h, in concentrations of IC_50_ and 2 IC_50_ values.

With the purpose of corroborating the mechanism of cellular death, flow cytometry studies were performed. Evaluation of their ability to promote cell death based on specific cell death markers, in particular, phosphatidylserine (PS) exposure on the outer face of the plasma membrane to detect apoptosis using Annexin V-DY634 as a marker, were conducted. As can be observed in Figure 6, both complexes induced apoptosis as cell death, and a more potent cytotoxic effect at higher concentrations was observed, especially for the compound silver species **PDI-3**.

### 3.5. Confocal Fluorescence Microscopy

Cell biodistribution of the ligand **PDI-2** and the metal complexes **PDI-3** and **PDI-4** was studied in HeLa cells. Quenching of the luminescent properties in the tetra-metallic complexes precluded the analysis of the biodistribution in cancer cells. A colocalization assay was performed where the ligand **PDI-2** and the copper complex **PDI-4** were incubated with HeLa cells together with a commercially available selective dye for a specific organelle as internal standard. The superimposition of the images obtained from the internal standard with those of the study compounds provides the cellular internalization of the compounds.

**Figure 7:**
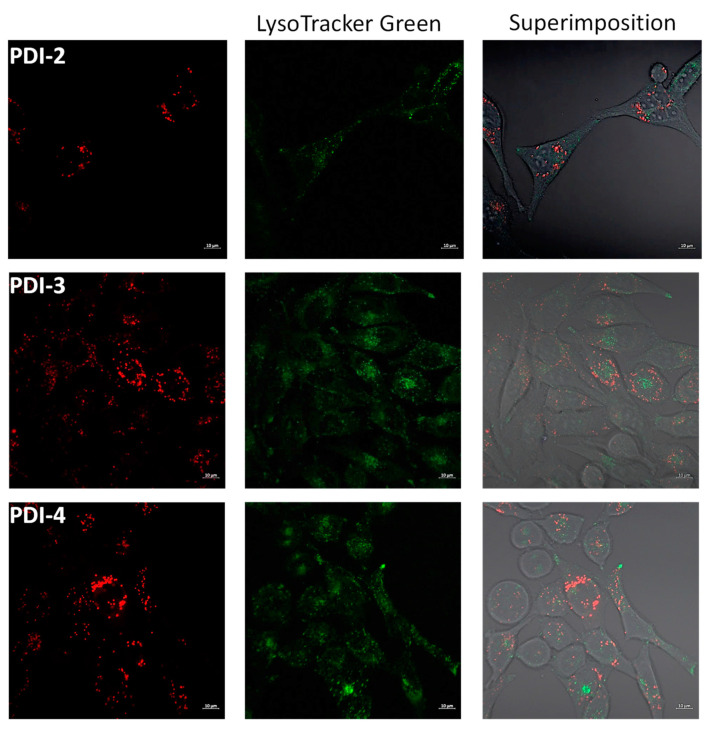
Fluorescence confocal microscopy images in HeLa cells incubated with the ligand **PDI-2** and the **PDI-3** and **PDI-4** complexes at 2 h (red, irradiated at 561 nm) and stained with LysoTracker Green (green, irradiation at 488 nm).

As many of these small molecules enter the cell with a passive transport and localize in the lysosomes, the colocalization experiment was performed using the LysoTracker Green with a different emission energy from the compounds. Figure 7 shows the emission inside the cells of the ligand and copper complexes in red, and in green the emission of the LysoTracker and the superimposition images, observing a slightly different emission pattern for each compound.

The **PDI-2** ligand presents a lower internalization inside the cells than the corresponding metal complexes, and all of them spread through the cytoplasm of the cell, non-entering in the nucleus. It can be observed in the superimposition images that neither the ligand or the complexes colocalized with the signal emitted by LysoTracker, indicating the absence of a lysosomal localization.

**Figure 8:**
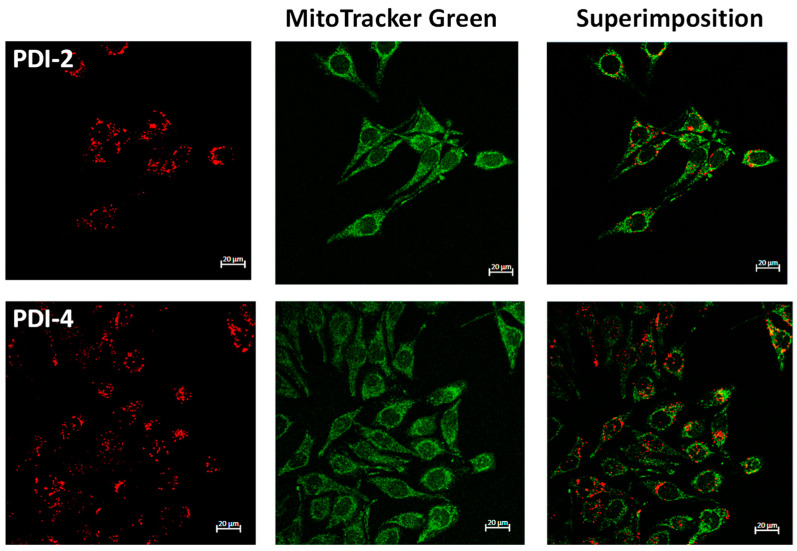
Fluorescence confocal microscopy images in HeLa cells incubated with the ligand **PDI-2** and the copper complex **PDI-4** at 2 h (red, irradiated at 561 nm) and stained with MitoTracker Green (green, irradiation at 488 nm).

In an attempt to elucidate the biodistribution and considering the previous experiment, compounds were incubated in HeLa cells for 2 h and MitoTracker Green, a mitochondrial selective dye, was added as internal standard. Mitochondria is an important biological target and several metal complexes targeting mitochondria have been encountered. Superimposition of the images reveals a partial mitochondrial localization for the ligand **PDI-2** and the copper complex **PDI-4** (Figure 8). Additionally, small spots near the nuclear region that do not match with the mitochondrial biodistribution can be observed. This accumulation may point to a localization in the Golgi apparatus, although further experiments with this specific dye as internal standard should be performed.

## 4. Conclusions

We report the synthesis of perylenediimide (PDI) derivatives bearing one or four dipyridylamino fragments with the purpose of studying the coordination properties to silver and copper phosphine complexes. These ligands coordinate to the metal fragments in a chelate fashion and mononuclear or tetranuclear complexes have been achieved. As perylenediimides are very interesting chromophore groups, the photophysical properties of the ligands and complexes have been studied. The coordination of the metal center did not reveal a significant effect on the emission energy in the complexes, which are mainly based on the PDI ligands, although a higher quantum yield was observed upon coordination of the metal complexes. For the tetranuclear silver or copper derivatives, quenching of the luminescence was observed.

The antiproliferative effect of the free perylenediimide ligand and the metal complexes against the cervix cancer cell line HeLa was determined by the MTT assay. The free perylenediimide ligands exhibit a moderate cytotoxic activity, but the coordination of silver or copper to the dypyridylamino fragment greatly enhanced the activity, suggesting a synergistic effect between the two fragments. Flow cytometry experiments showed that the metal complexes induce an apoptotic cell death. To assert the cellular biodistribution of the PDIs and the complexes, a colocalization experiment using specific dyes for the lysosomes or mitochondria as internal standards revealed a major internalization inside the cell for the metal complexes as well as a partial mitochondrial localization.

## Figures and Tables

**Table 1:** IC_50_ values of **PDI-2**–**PDI-**4 and **PDI-6**–**PDI-**8 incubated for 24 h in HeLa cells.

Compound	IC_50_ (µM)
**PDI-2**	11.51 ± 0.9
**PDI-3**	2.46 ± 0.1
**PDI-4**	3.08 ± 0.6
**PDI-6**	10.54 ± 0.8
**PDI-7**	2.05 ± 0.9
**PDI-8**	1.90 ± 0.1

## Data Availability

Not applicable.

## Supplementary Materials

The following supporting information can be downloaded at: https://www.mdpi.com/article/10.3390/pharmaceutics14122616/s1. Figure S1: ^1^H NMR spectrum of PDI-2. Figure S2: ^13^C NMR spectrum of PDI-2. Figure S3: MALDI-TOF spectrum of PDI-2. Figure S4: UV-Vis and fluorescence spectra of PDI-2. Figure S5: IR spectrum (KBr) of PDI-2. Figure S6: ^1^H NMR spectrum of PDI-6. Figure S7: 1^3^C NMR spectrum of PDI-6. Figure S8: MALDI-TOF spectrum of PDI-6. Figure S9: UV-Vis and fluorescence spectra of PDI-6. Figure S10: IR spectrum (KBr) of PDI-6. Figure S11: ^1^H NMR spectrum of PDI-3. Figure S12: MALDI-TOF spectrum of PDI-3. Figure S13: IR spectrum (KBr) of PDI-3. Figure S14: ^1^H NMR spectrum of PDI-4. Figure S15: MALDI-TOF spectrum of PDI-4. Figure S16: IR spectrum (KBr) of PDI-4. Figure S17: ^1^H NMR spectrum of PDI-7. Figure S18: IR spectrum (KBr) of PDI-7. Figure S19: ^1^H NMR spectrum of PDI-8. Figure S20: IR spectrum (KBr) of PDI-8. Figure S21: UV-Vis spectra of PDI-complexes. Figure S22: Fluorescence spectra of PDI-3 and PDI-4. Figure S23: UV-Vis spectra of PDI-2, -3, -7, -8 in PBS solution + 5% DMSO at 37.5 °C, at 0 and 24 h. Figure S24: Dose–response curves of HeLa cells after incubation with cationic PDI-2, -3, -4, and PDI-6, -7, -8 for 24 h.
