# Supplementary material for: Synthesis of Dipyridylaminoperylenediimide–Metal Complexes and Their Cytotoxicity Studies
Source: Pharmaceutics. 2022 Nov 27;14(12):2616. doi: 10.3390/pharmaceutics14122616 (PMC9781374; doi:10.3390/pharmaceutics14122616)
Supplement: Supplementary file 1 [file pharmaceutics-14-02616-s001.zip › pharmaceutics-2042453-supplementary.pdf]

Supplementary Data

Synthesis of Dipyridylaminoperylenediimide–Metal Complexes and Their Cytotoxicity Studies

Table of Contents

- Figure S1: ^{1}H NMR spectrum of PDI-2 Pag 2
- Figure S2: ^{13}C NMR spectrum of PDI-2 Pag 3
- Figure S3: MALDI-TOF spectrum of PDI-2 Pag 3
- Figure S4: UV-Vis and fluorescence spectra of PDI-2 Pag 4
- Figure S5: IR spectrum (KBr) of PDI-2 Pag 4
- Figure S6: ^{1}H NMR spectrum of PDI-6 Pag 5
- Figure S7: ^{13}C NMR spectrum of PDI-6 Pag 5
- Figure S8: MALDI-TOF spectrum of PDI-6 Pag 6
- Figure S9: UV-Vis and fluorescence spectra of PDI-6 Pag 6
- Figure S10: IR spectrum (KBr) of PDI-6 Pag 7
- Figure S11: ^{1}H NMR spectrum of PDI-3 Pag 7
- Figure S12: MALDI-TOF spectrum of PDI-3 Pag 8
- Figure S13: IR spectrum (KBr) of PDI-3 Pag 8
- Figure S14: ^{1}H NMR spectrum of PDI-4 Pag 9
- Figure S15: MALDI-TOF spectrum of PDI-4 Pag 9
- Figure S16: IR spectrum (KBr) of PDI-4 Pag 10
- Figure S17: ^{1}H NMR spectrum of PDI-7 Pag 10
- Figure S18: IR spectrum (KBr) of PDI-7 Pag 11
- Figure S19: ^{1}H NMR spectrum of PDI-8 Pag 11
- Figure S20: IR spectrum (KBr) of PDI-8 Pag 12
- Figure S21: UV-Vis spectra of PDI-complexes Pag 13
- Figure S22: Fluorescence spectra of PDI-3 and PDI-4 Pag 13
- Figure S23: UV-Vis spectra of PDI-2, -3, -7, -8 in PBS solution + 5% DMSO at 37.5 ºC, at 0 and 24 h. Pag 14
- Figure S24: Dose-response curves of HeLa cells after incubation with cationic PDI-2, -3, -4, and PDI-6, -7, -8 for 24 hours. Pag 15

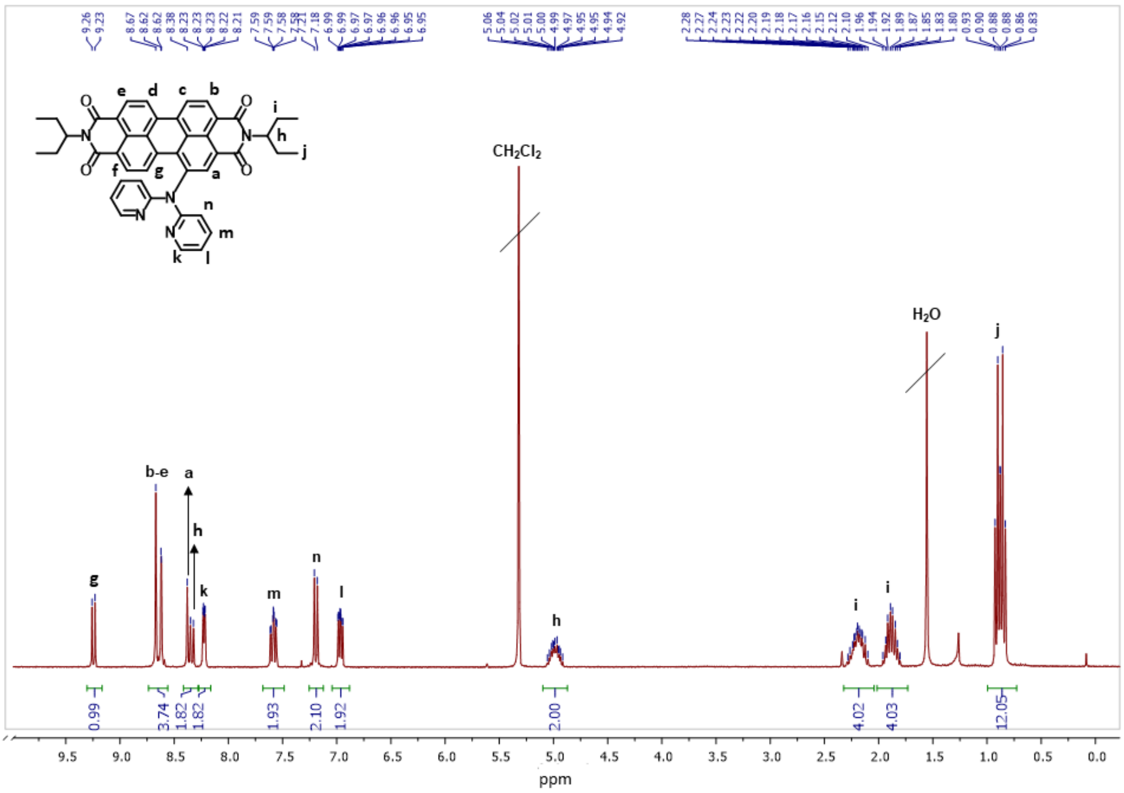

Figure S1: ^{1}H NMR spectrum of PDI-2 in CD_2Cl_2 (300 MHz, 25 ºC)

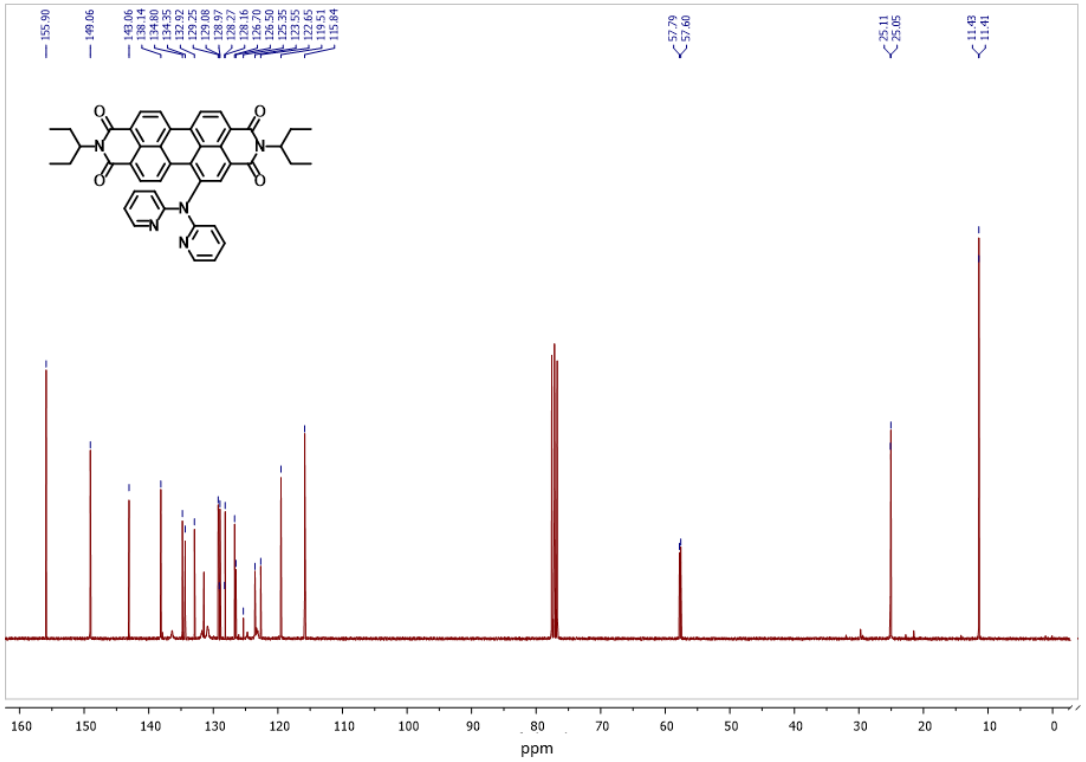

Figure S2: ^{13}C NMR spectrum of PDI-2 in $CDCl_3$ (75 MHz, 25 ^{o}C)

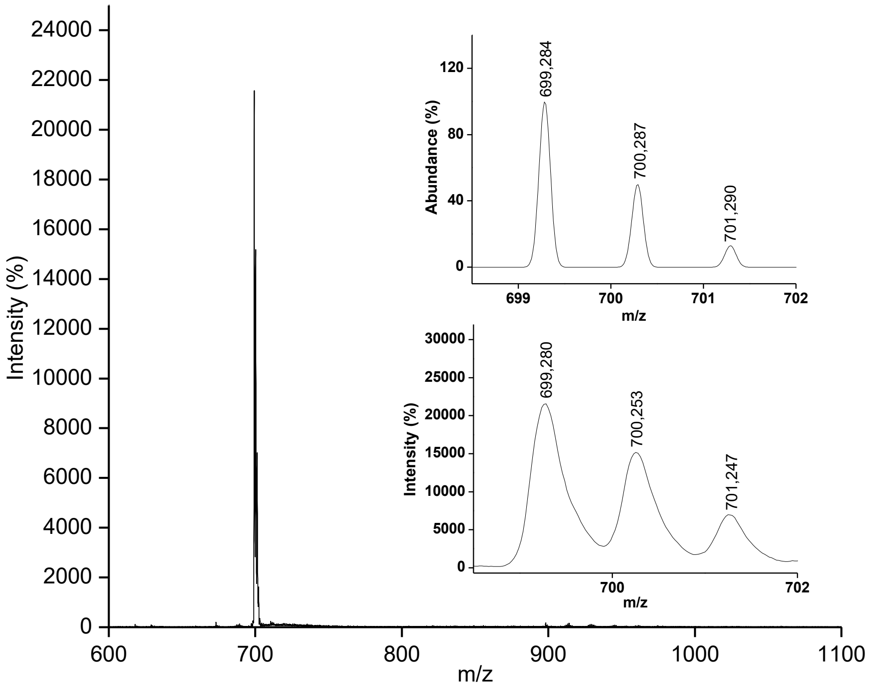

Figure S3: MALDI-TOF spectrum of PDI-2

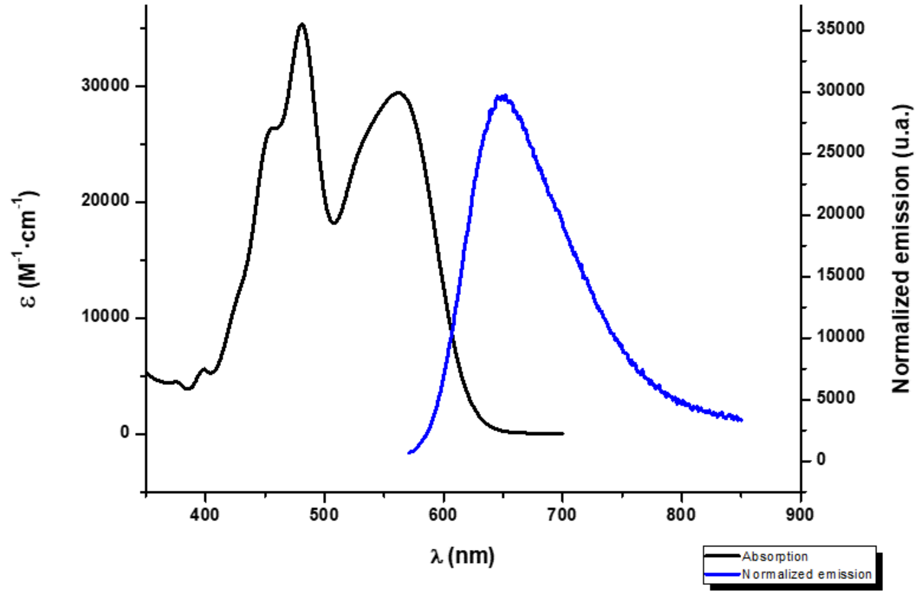

Figure S4: UV-Vis and fluorescence spectra of PDI-2

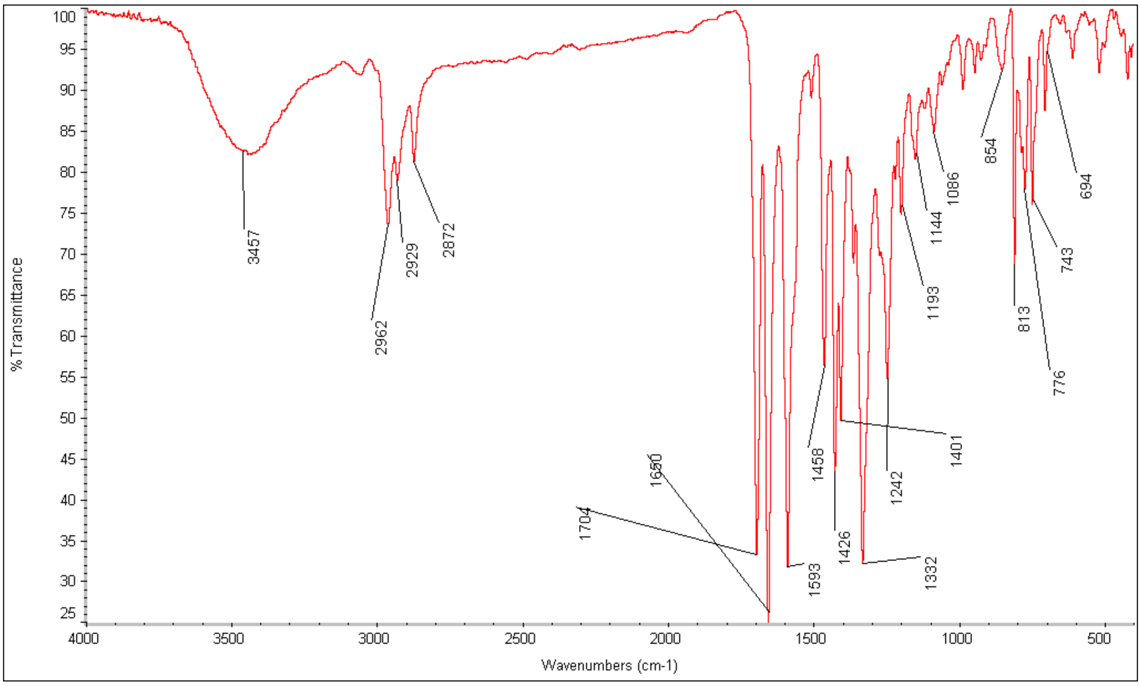

Figure S5: IR spectrum (KBr) of PDI-2

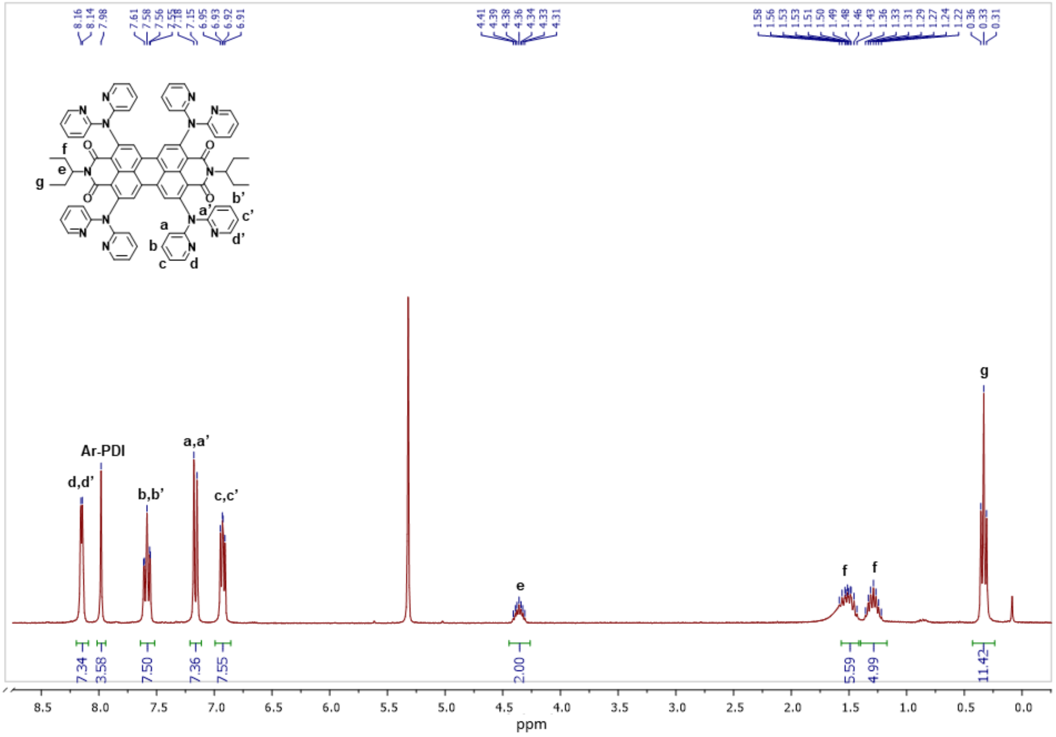

Figure S6: ^{1}H NMR spectrum of PDI-6 in CD_2Cl_2 (300 MHz, 25 °C)

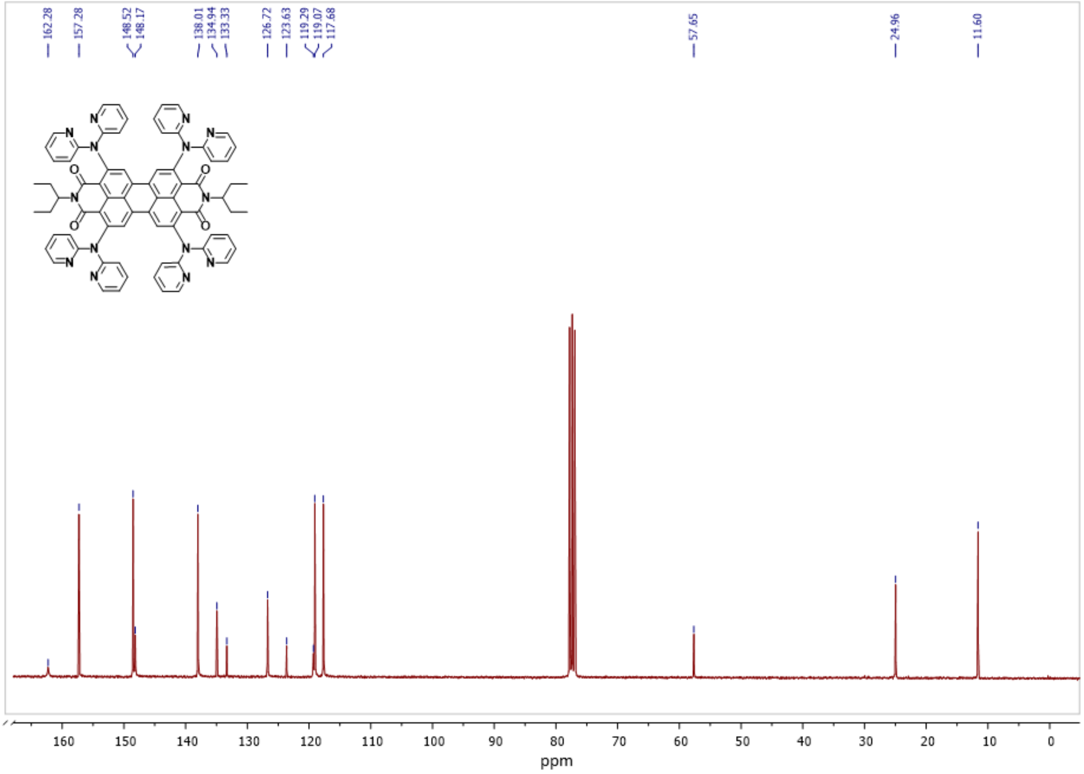

Figure S7: ^{13}C NMR spectrum of PDI-6 in $CDCl_3$ (75 MHz, 25 °C)

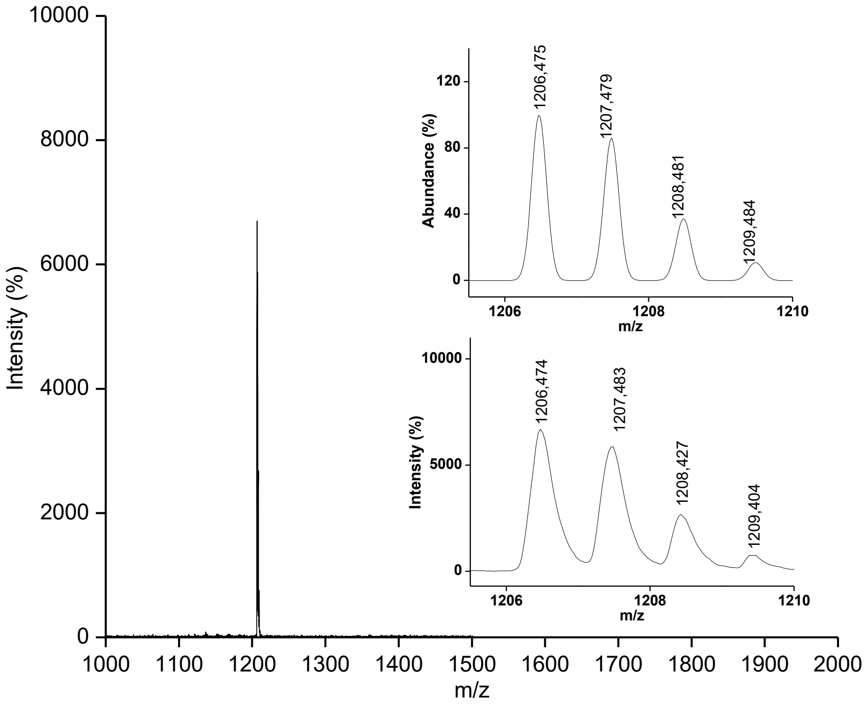

Figure S8: MALDI-TOF spectrum of PDI-6

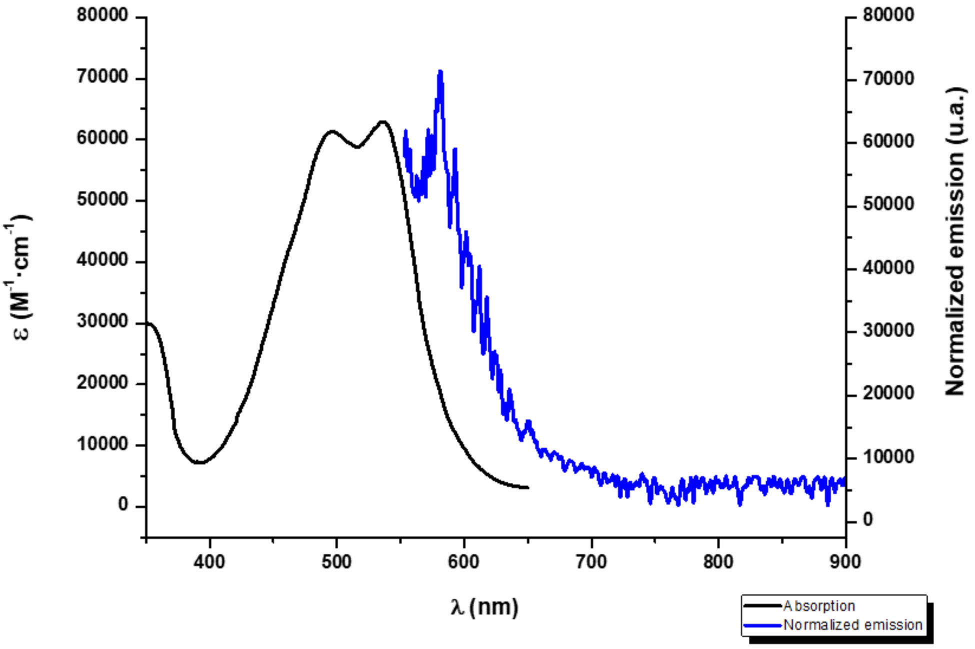

Figure S9: UV-Vis and fluorescence spectra of PDI-6

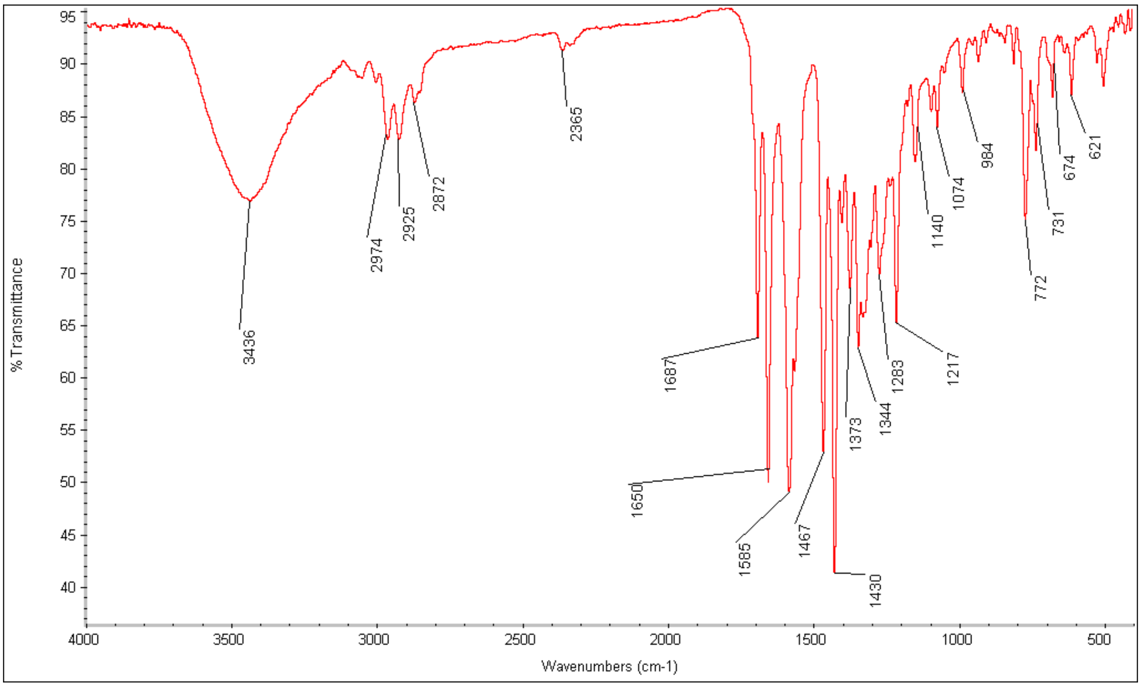

Figure S10: IR spectrum (KBr) of PDI-6

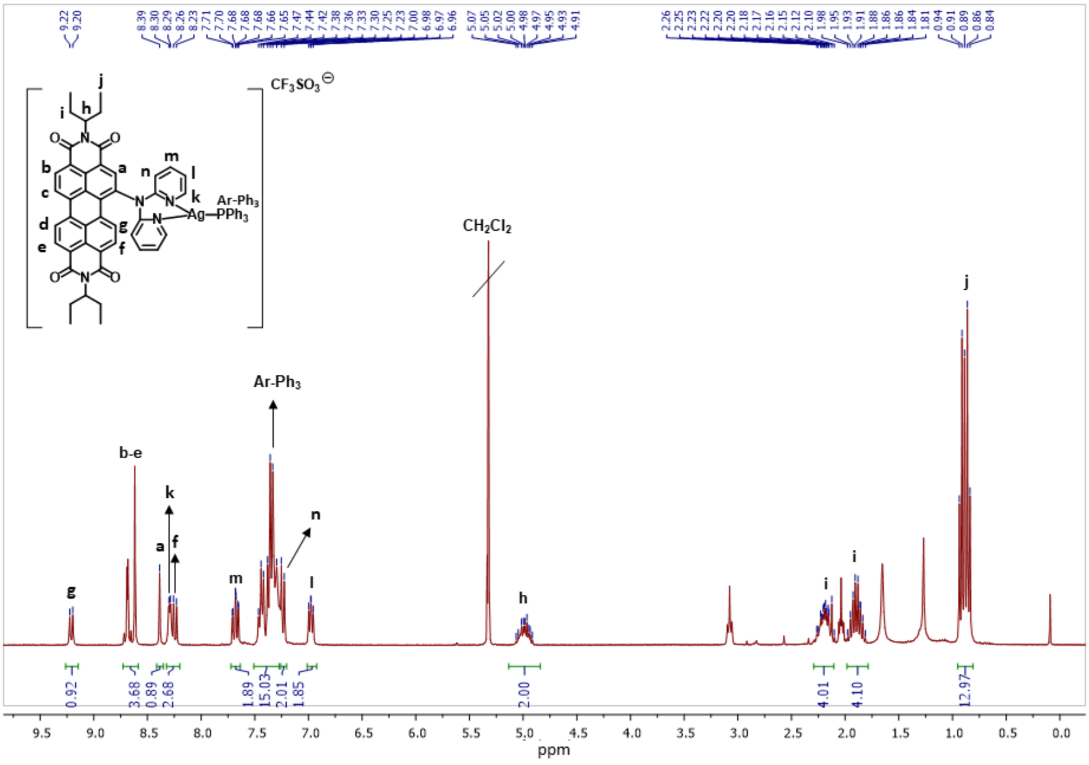

Figure S11: ^{1}H NMR spectrum of PDI-3 in CD_2Cl_2 (300 MHz, 25 °C)

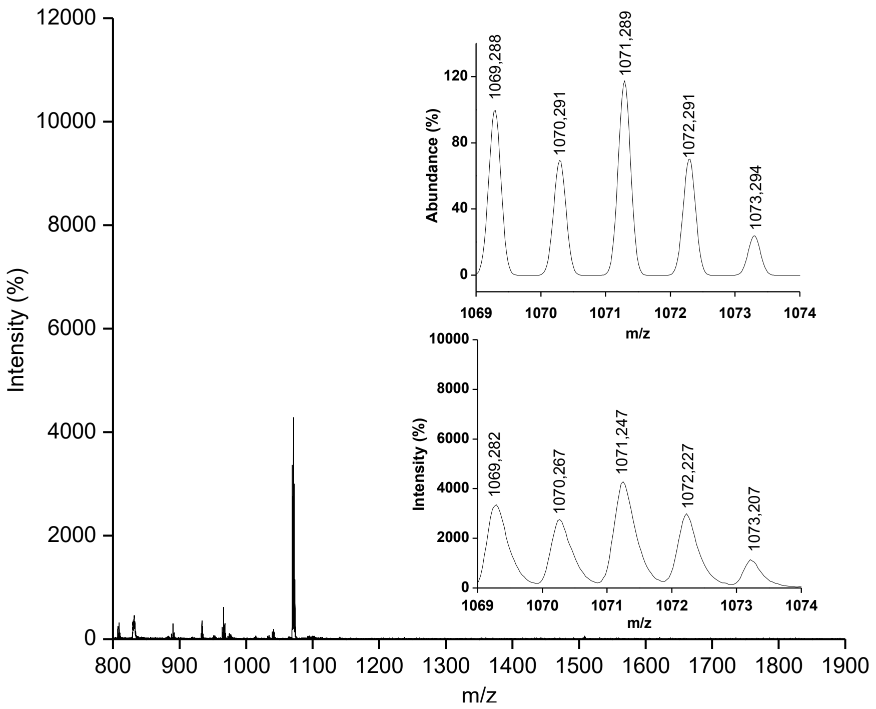

Figure S12: MALDI-TOF spectrum of PDI-3

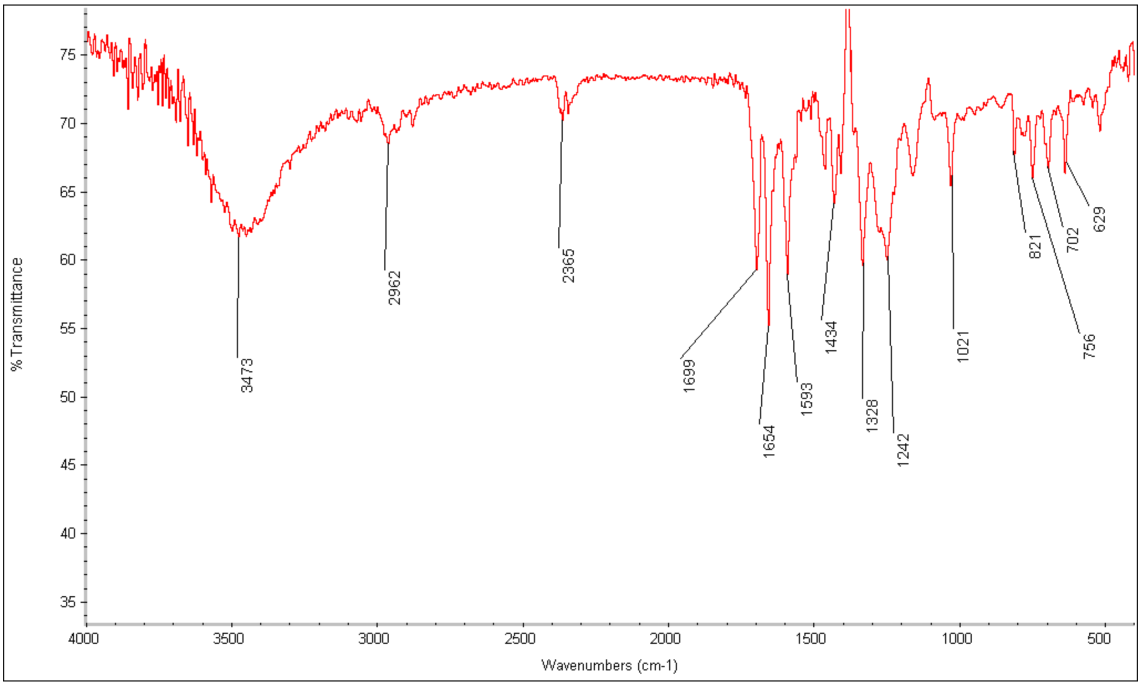

Figure S13: IR spectrum (KBr) of PDI-3

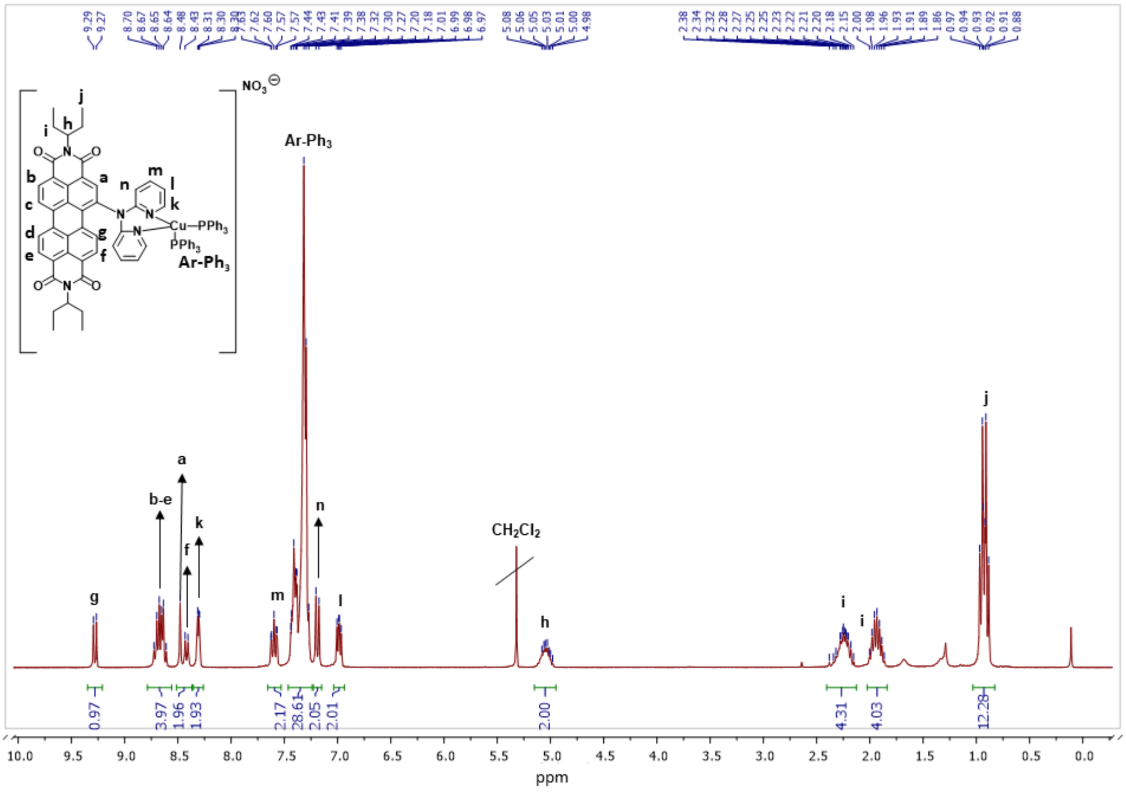

Figure S14: ^{1}H NMR spectrum of PDI-4 in CD_2Cl_2 (300 MHz, 25 °C)

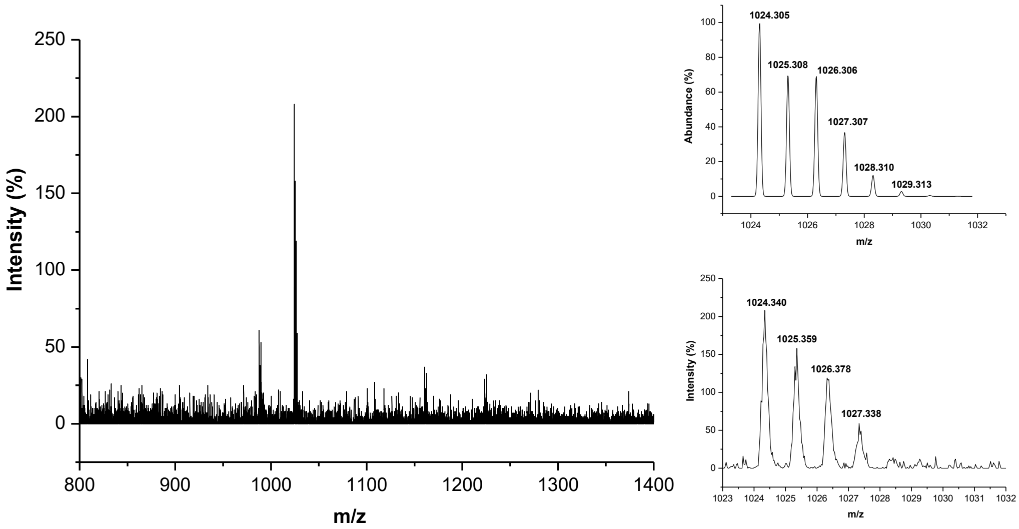

Figure S15: MALDI-TOF spectrum of PDI-4

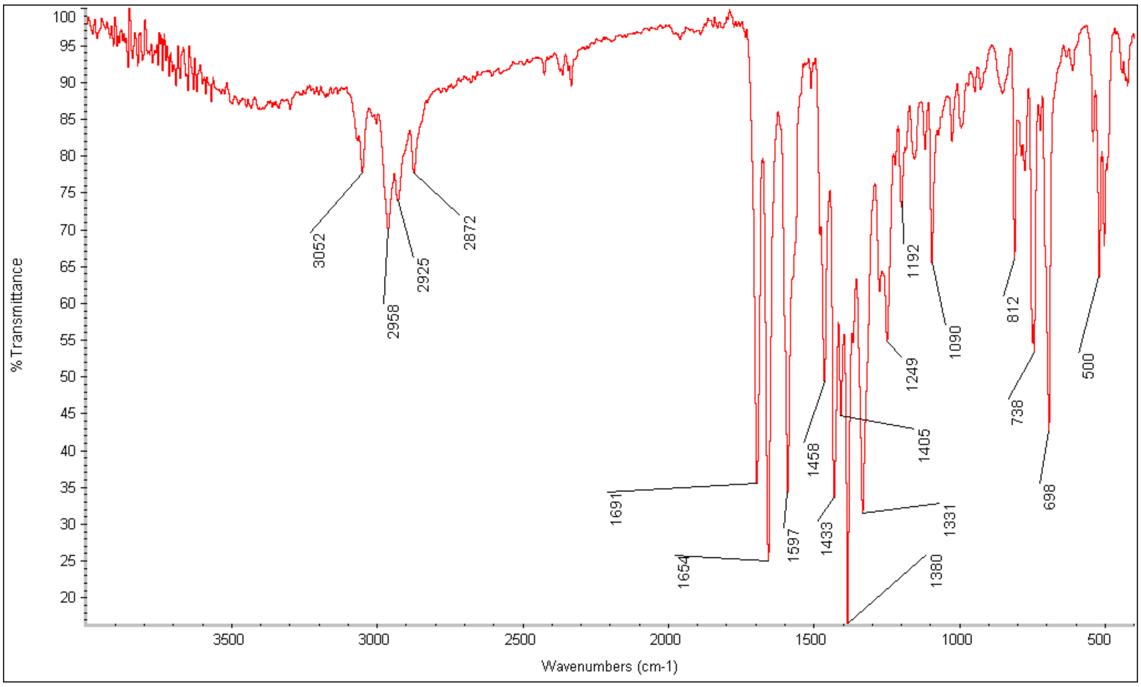

Figure S16: IR spectrum (KBr) of PDI-4

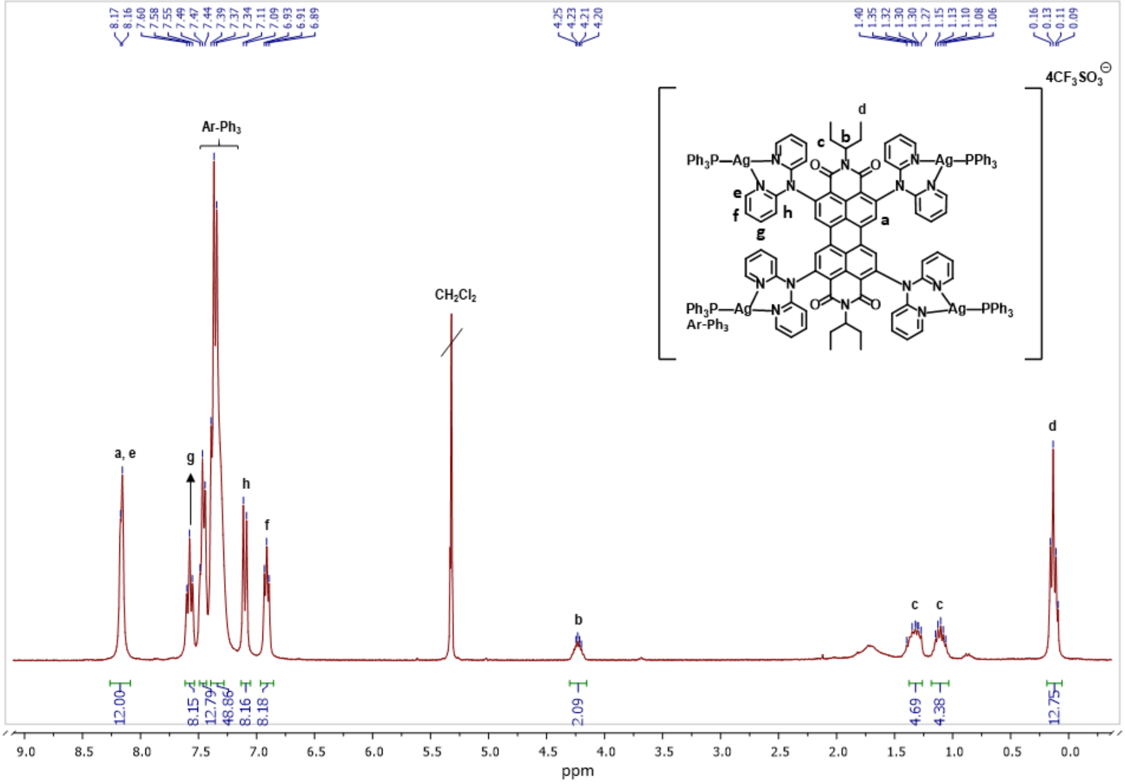

Figure S17: ^{1}H NMR spectrum of PDI-7 in CD_2Cl_2 (300 MHz, 25 °C)

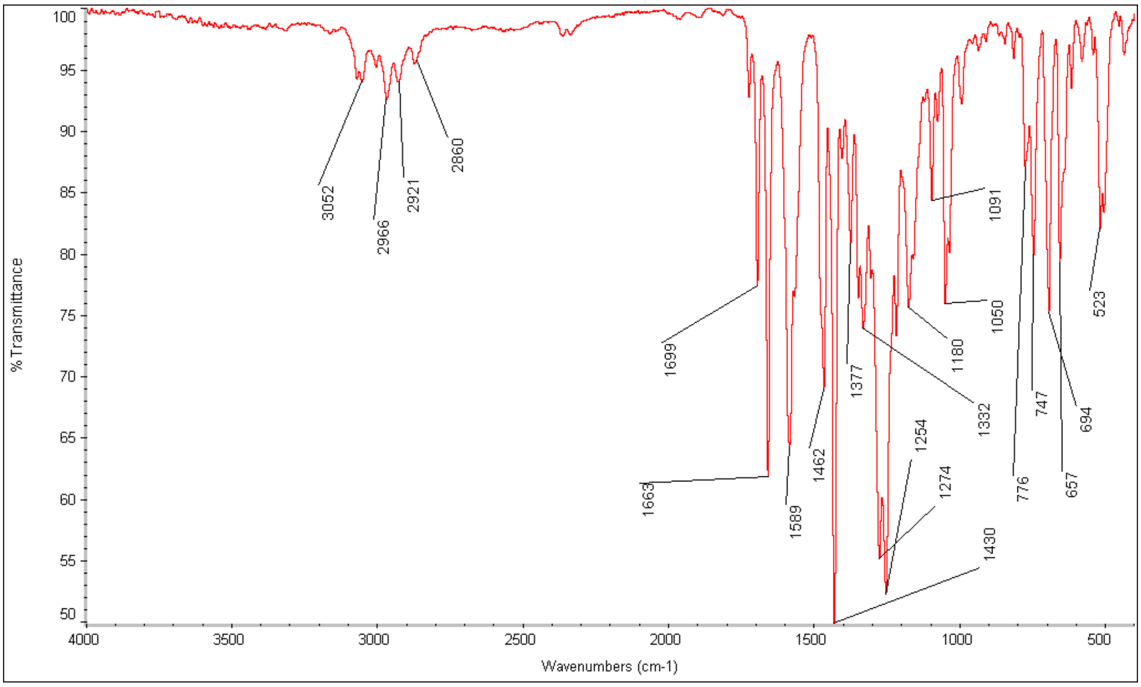

Figure S18: IR spectrum (KBr) of PDI-7

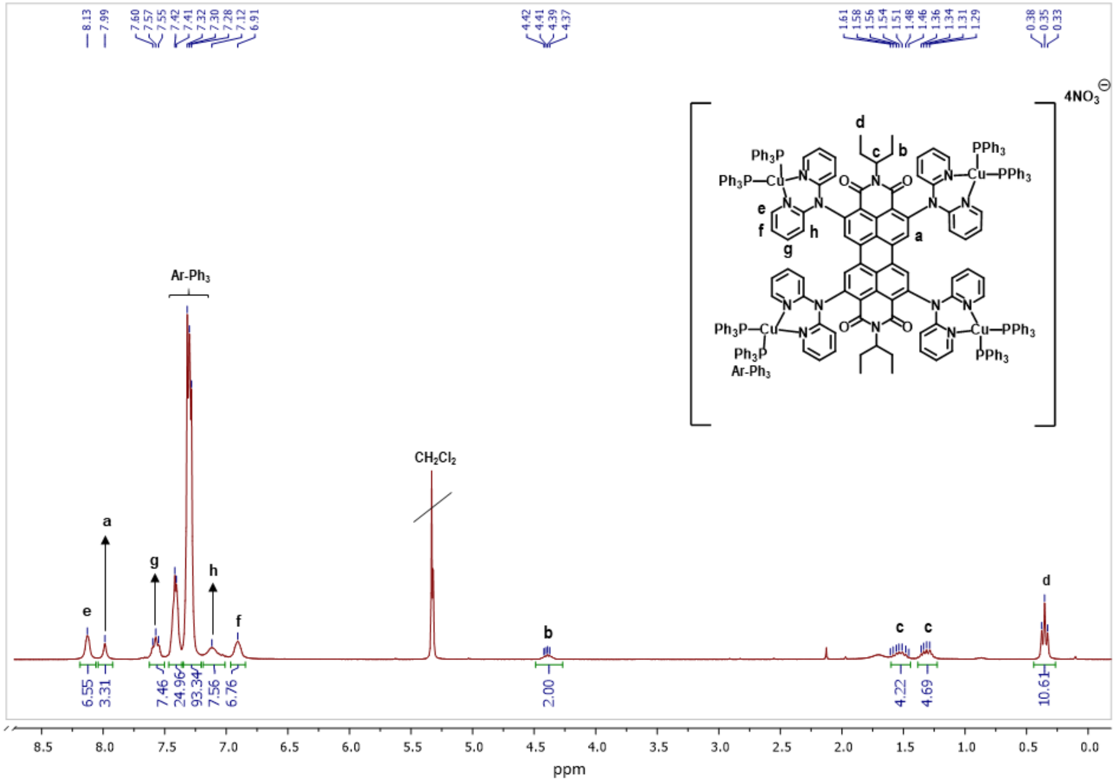

Figure S19: ^{1}H NMR spectrum of PDI-8 in CD_2Cl_2 (300 MHz, 25 °C)

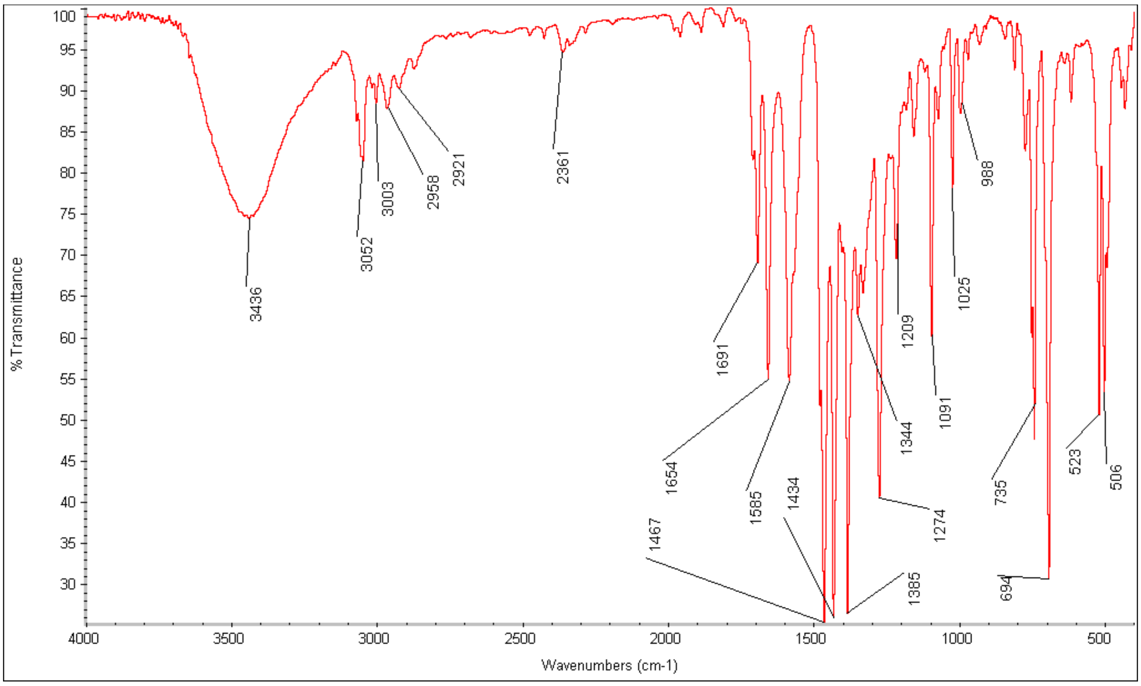

Figure S20: IR spectrum (KBr) of PDI-8

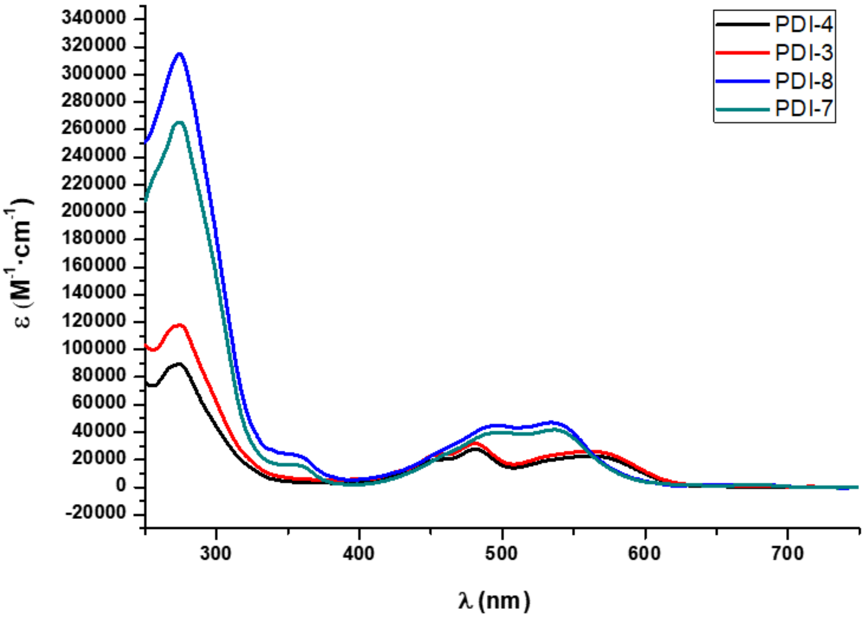

Figure S21: UV-Vis spectra of PDI-complexes ($CHCl_3$)

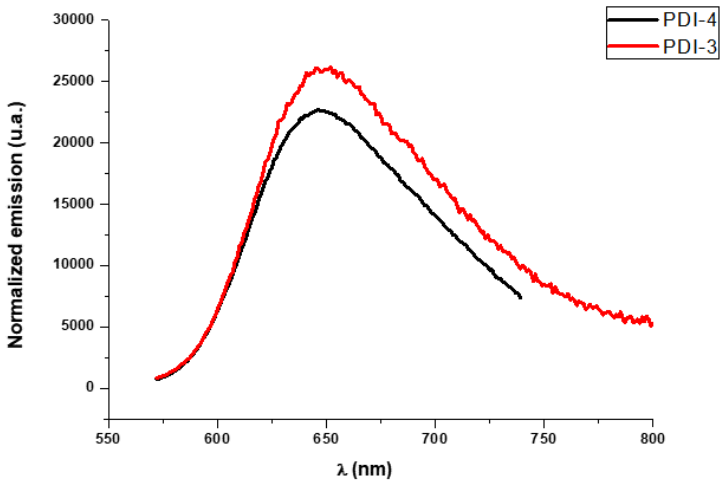

Figure S22: Fluorescence spectra of PDI-3 and PDI-4 ($CHCl_3$)

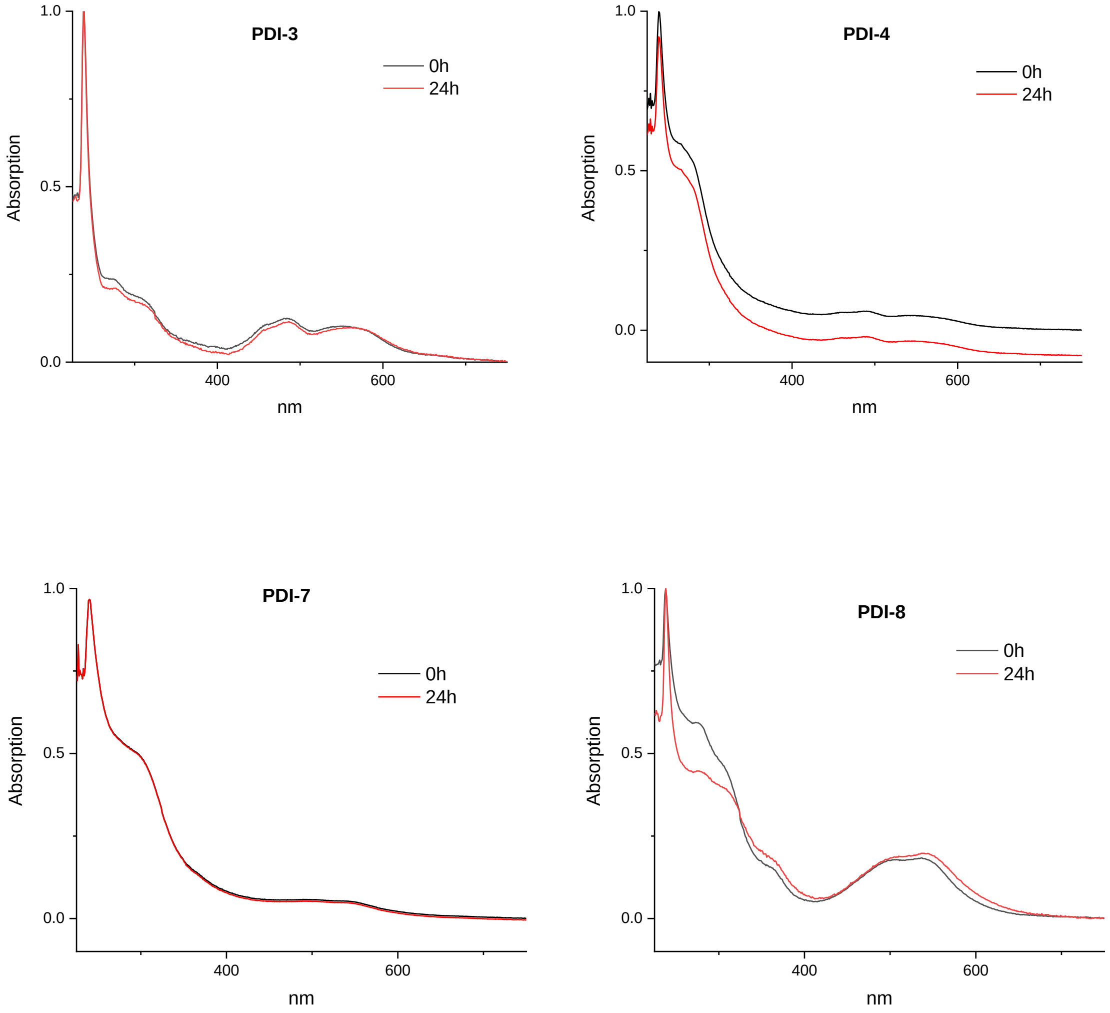

Figure S23: UV-Vis spectra of PDI-2, -3, -7, -8 in PBS solution + 5% DMSO at 37.5 ºC, at 0 and 24 h (concentration 5·10^{-4}).

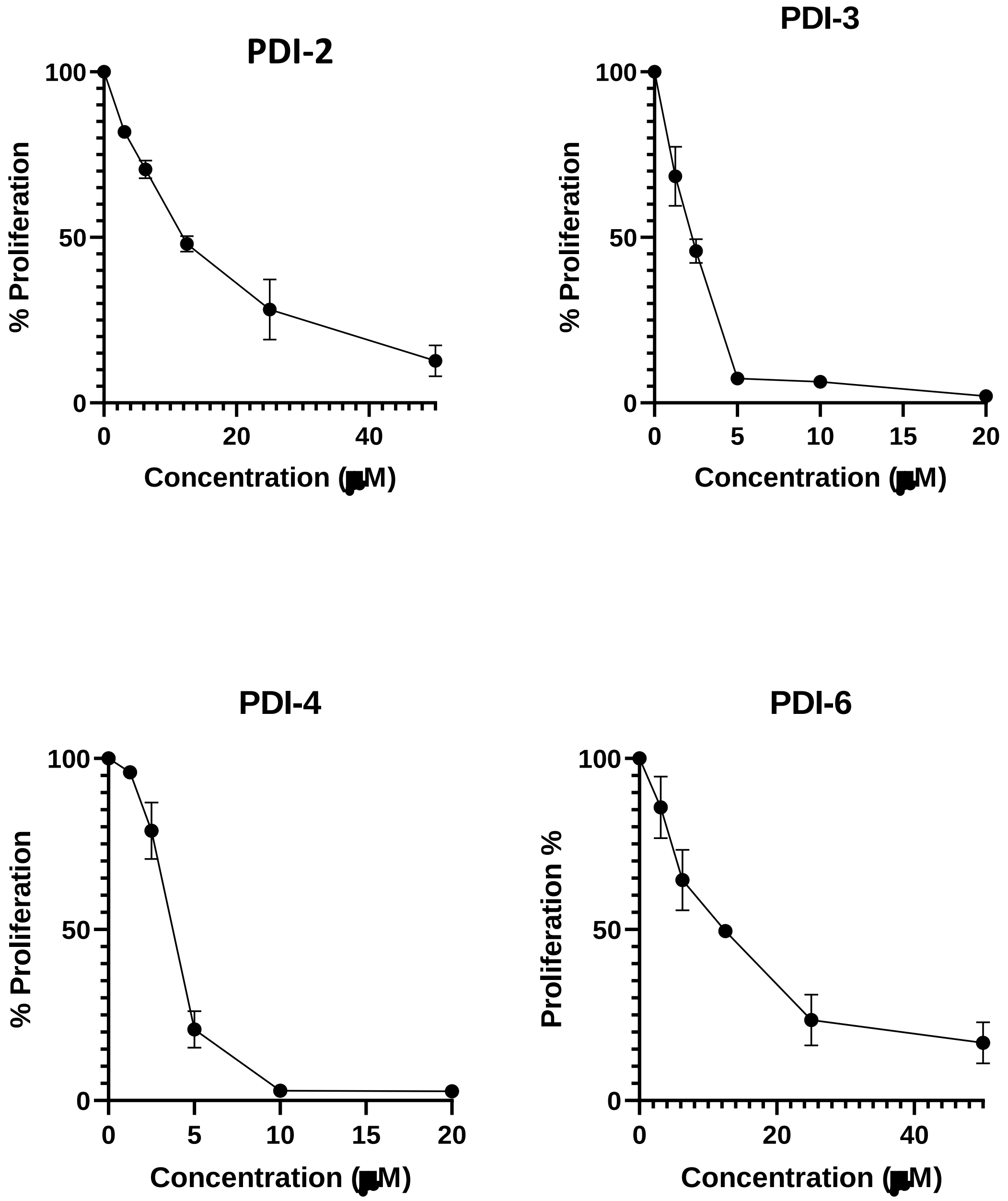
PDI-2
100
50
0
% Proliferation
0
20
40
Concentration (µM)
PDI-3
100
50
0
% Proliferation
0
5
10
15
20
Concentration (µM)
PDI-4
100
50
0
% Proliferation
0
5
10
15
20
Concentration (µM)
PDI-6
100
50
0
Proliferation %
0
20
40
Concentration (µM)

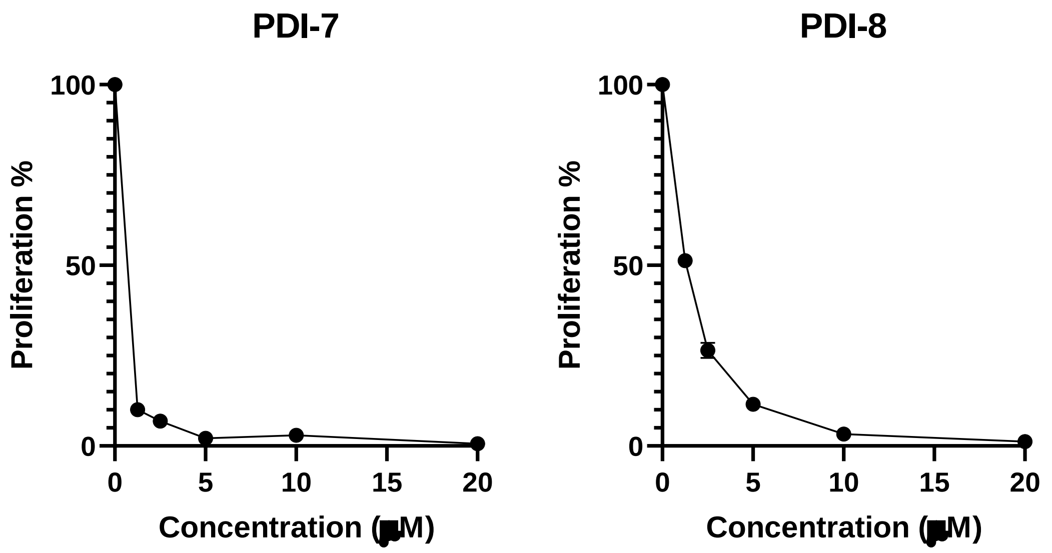

Figure S24: Dose-response curves of HeLa cells after incubation with cationic PDI-2, -3, -4, and PDI-6, -7, -8 for 24 hours.
